# MYD88 signals induce tumour-initiating cell generation through the NF-κB-HIF-1α activation cascade

**DOI:** 10.1038/s41598-021-83603-4

**Published:** 2021-02-17

**Authors:** Atsuko Tanimura, Akane Nakazato, Nobuyuki Tanaka

**Affiliations:** Department of Molecular Oncology, Institute for Advanced Medical Sciences, Nippon Medical School, Tokyo, Japan

**Keywords:** Cancer stem cells, Cancer microenvironment

## Abstract

Tumour-promoting inflammation is a hallmark of cancer, and chronic inflammatory disease increases the risk of cancer. In this context, MYD88, a downstream signalling molecule of Toll-like receptors that initiates inflammatory signalling cascades, has a critical role in tumour development in mice and its gene mutation was found in human cancers. In inflammation-induced colon cancer, tumour suppressor p53 mutations have also been detected with high frequency as early events. However, the molecular mechanism of MYD88-induced cancer development is poorly understood. Here, we demonstrated that MYD88 induced the protein accumulation of the transcription factor HIF-1α through NF-κB in p53-deficient cells. HIF-1α accumulation was not caused by enhanced protein stability but by NF-κB-mediated transcriptional activation, the enhanced translation of HIF-1α and JNK activation. In contrast, MYD88-induced mRNA expressions of HIF-1α and HIF-1-target genes were attenuated in the presence of p53. Furthermore, constitutively active forms of MYD88 induced tumour-initiating cell (TIC) generation in p53-deficient cells, as determined by tumour xenografts in nude mice. TIC generating activity was diminished by the suppression of NF-κB or HIF-1α. These results indicate that MYD88 signals induce the generation of TICs through the NF-κB-HIF-1α activation cascade in p53-deficient cells and suggest this molecular mechanism underlies inflammation-induced cancer development.

## Introduction

Tumour-initiating cells (TICs), also called cancer stem cells, are a subset of tumour cells that have self-renewal properties, tumour initiation capacity, and long-term tumour repopulation potential^1,2^. The term “cancer-initiating” has been used to refer to the ability of cells to form tumours when transplanted into immunodeficient mice such as nude mice^3^. Accumulating evidence has suggested that the molecular mechanisms that underlie the generation of induced pluripotent stem cells (iPSCs) have parallels with the cancer initiation process^4^. For example, the transient in vivo expression of reprogramming factors required for iPSC generation, octamer-binding transcription factor 3/4 (OCT3/4), Krüppel-like factor 4 (KLF4), SRY-box 2 (SOX2), and MYC, results in tumour development in various tissues containing undifferentiated cells with global changes in DNA methylation patterns^5^. These findings suggest that the induction of reprogramming factors and subsequent epigenetic regulation induce TIC generation.

Studies have shown that gene-expression signatures specific to cancer and normal stem cells are significantly related to the treatment outcome of patients with diverse driver mutations, suggesting that stemness is a central biological property or process upon which many driver mutations converge^6^. Moreover, cytotoxic agents such as radiation and chemotherapy efficiently target most types of cancer cells and are commonly used to treat cancer; however, in the clinic, TICs often show resistance to such therapies. Therefore, residual cancer tissues can be enriched in TIC populations that trigger tumour recurrence^2^. Taken together, it is important to elucidate the signal cascade that leads to the induction of reprogramming factors and epigenetic regulation associated with the generation of TICs during oncogenesis to develop effective cancer treatments, especially for the prevention of cancer recurrence. Therefore, we investigated the signal cascade necessary for TIC production in inflammation-induced cancers as a model of cancer development^7^.

The innate immune response, the first line of defence against pathogens, consists of pattern-recognition receptors, such as Toll-like receptors (TLRs) that express on the cell surface and recognise pathogen-specific structures^8,9^. TLRs contain an ectodomain that mediates the recognition of unique microbial molecules (so-called pathogen-associated molecular patterns; PAMPs), a transmembrane domain, and cytosolic Toll/interleukin-1 (IL-1) receptor (TIR) domains that mediate activation of intracellular signalling pathways^9,10^. The TLR signals are transmitted by adaptor molecules that harbour a TIR domain, myeloid differentiation primary response protein 88 (MYD88), and TIR domain-containing adaptor protein inducing interferon beta (TRIF), which induce the secretion of type I interferons, inflammatory cytokines/chemokines and antimicrobial peptides. MYD88 was originally identified as a protein induced during IL-6-induced terminal differentiation of myeloid precursor cells. The critical role of MYD88 in TLR-signals was determined using MYD88-deficient mice^11^. MYD88 interacts with IL-1 receptor-associated kinase (IRAK) family proteins, especially IRAK4, and these IRAKs activate downstream kinases including c-Jun N-terminal kinase (JNK) and p38, and transcription factors such as nuclear factor-κB (NF-κB)^11^.

The crosstalk between inflammation and cancer development has been demonstrated by experimental animal model and its underlying molecular mechanism has been analysed^12,13^, indicating that inflammation can reprogram cell fate to TICs. In this context, MYD88 has also been shown to play an integral role in spontaneous tumour development in mice with a mutation in the adenomatous polyposis coli (APC) gene and in carcinogen-induced colon tumour development^14^. Furthermore, MYD88 was shown to regulate the expression of cyclooxygenase 2, matrix metalloproteinase 7, and cytosolic phospholipase A2^14^, which are important for tumour growth^15–17^. In addition, epithelial barrier deterioration induced by colorectal-cancer-initiating genetic lesions resulted in the invasion of microbes that triggered tumour-elicited inflammation, resulting in enhanced tumour growth in APC mice^18^. Therefore, tumour-elicited inflammation and TLR-MYD88 signals provoked by the infection of microbes induce the generation of TICs with cancer-prone genetic alterations.

In addition to their role in inflammation-induced cancers, it was demonstrated that 29% of activated B-cell-like subtype of diffuse large B-cell lymphomas harboured an amino acid substitution, L265P, in the MYD88 TIR domain, and that some rare cases had other mutations^19^. Survival of lymphoma cells bearing the L265P mutation was sustained by mutant MYD88, demonstrating that L265P is a gain-of-function driver mutation. The L265P mutant promoted cell survival by assembling a protein complex containing IRAK1 and IRAK4, leading to the activation of IRAK4 kinase, followed by the activation of NF-κB and its downstream signal cascade^19^. Moreover, in most cases of Waldenström’s macroglobulinemia, a low-grade B-cell neoplasm that invades bone marrow and secretes monoclonal IgM, MYD88 L265P mutations were observed^20^, indicating MYD88 enhances tumour development as well as generating TICs. However, the signal cascade of MYD88 related to the generation of TICs is unclear.

Patients with inflammatory bowel diseases (IBD) such as Crohn’s disease (CD) and ulcerative colitis (UC) are at increased risk for colorectal cancers^21^, and genomic alterations in suppressor p53 occurred in 89% of IBD cases (CD 83% and UC 94%)^22^. In IBD patients, p53 mutations have also been shown as early events in colorectal cancer, even prior to the development of dysplasia^23^. Moreover, p53 mutations were found in areas of mucosal inflammation in IBD^24^. These findings suggest that inflammation promotes p53 gene mutations and that a functional deficiency of p53 enhances the risk of inflammation-induced oncogenesis. Furthermore, several studies have shown that p53 acts as a barrier against the reprogramming of somatic cells to stem cells^25–29^. p53-deficient mouse embryonic fibroblasts (MEFs) gave rise to iPSCs using only two reprogramming factors, OCT3/4 and SOX2^26^, and these cells acquired tumour-initiating ability in nude mice by a single oncogene^30^, suggesting that the loss of p53 changes the cell fate to TICs by oncogenic signals. Therefore, to understand the mechanism of inflammation-induced cancer development, we examined the tumour-initiating ability and underlying mechanism of MYD88 in p53-deficient MEFs.

## Results

### MYD88 induces glycolysis and activates protein accumulation of the transcription factor HIF-1α in p53-deficient cells

**Figure 1:**
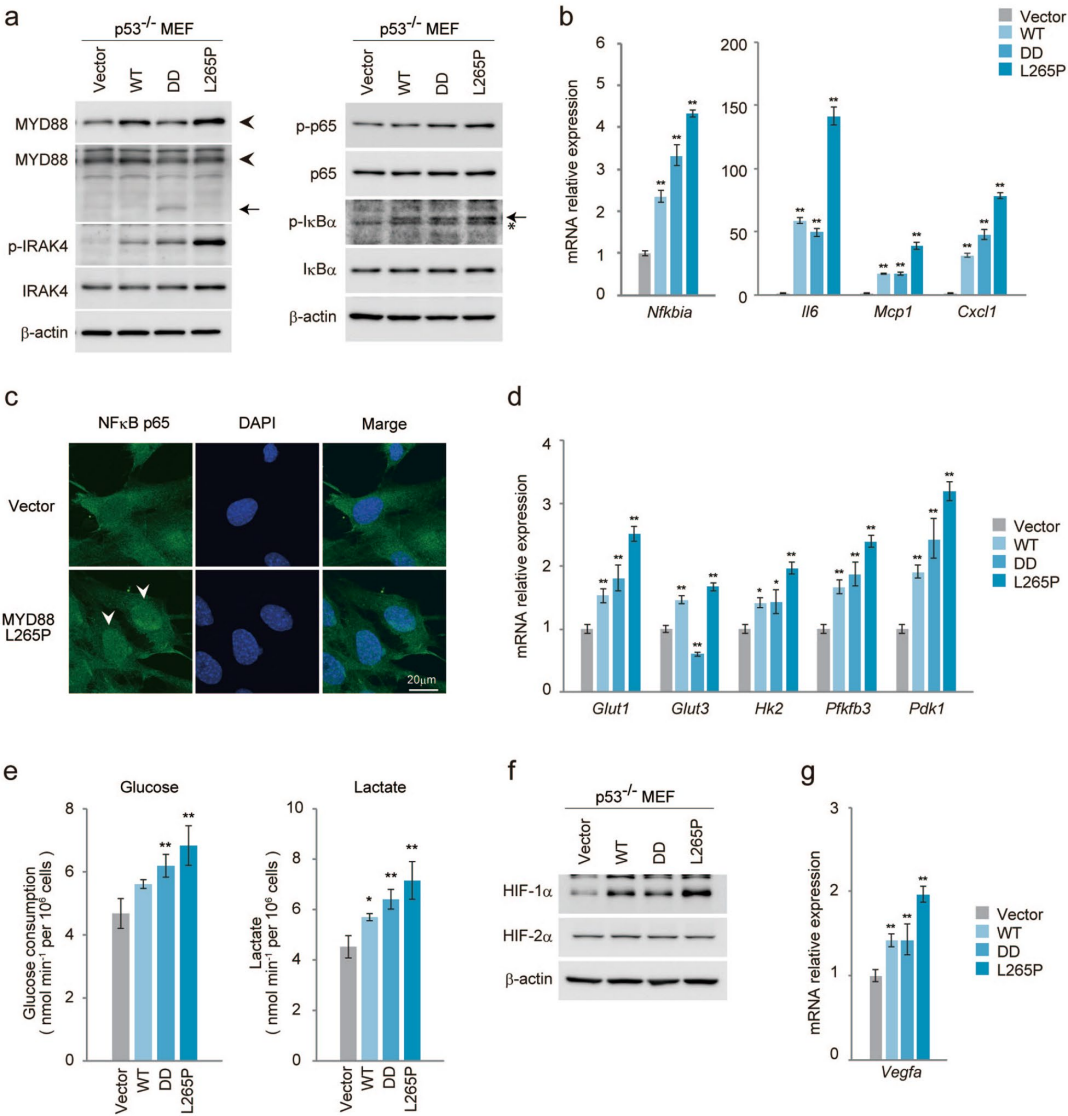
MYD88 signals activate NF-κB p65 and glucose metabolism in *p53*^*−/−*^MEFs. The indicated MYD88 constructs were introduced to *p53*^*−/−*^MEFs by retroviral infection. (**a**) Total cell lysate was analysed by immunoblotting. Left panel: The arrowhead indicates MYD88 and the arrow indicates truncated MYD88 (DD). Right panel: The arrow indicates phosphorylated-IκBα and the asterisk indicates a nonspecific band. All images of the original blots are shown in Fig. S9. (**b**) Quantitative polymerase chain reaction (qPCR) of NF-κB target genes. (**c**) Immunofluorescence staining shows NF-κB p65 nuclear localisation. Scale bar, 20 μm. (**d**) Expression of glucose metabolism-related gene mRNAs quantified by qPCR. (**e**) Glucose uptake and lactate production were measured. Statistical analysis was performed using one-way ANOVA followed by Scheffe’s F test. The quantified results are presented as the mean ± s.d. (n = 4). *P < 0.05, **P < 0.01 as compared with Vector. (**f**) Total cell lysates were analysed by immunoblotting. (**g**) Expression of *vegfa* mRNA was measured by qPCR. (**b**,**d**,**g**) The y-axis values are the relative fold change for gene transcripts normalised to β-actin. The data represent the mean ± s.d. (n = 3) using one-way ANOVA followed by Scheffe’s F test. *P < 0.05, **P < 0.01 as compared with Vector.

To elucidate the role of MYD88 in tumour development, we generated retroviral vectors expressing murine *Myd88* cDNA constructs including wild type (WT) and constitutively active forms of MYD88, a truncated form containing a death domain (DD)^31^ and a point mutant (L265P) corresponding to the human L265P mutation, which was identified as a gain-of-function driver mutation of human lymphoma^19^. As shown in Fig. 1a, p53-deficient MEFs (*p53*^*−/−*^MEFs) expressing MYD88 WT, DD or L265P, but not control vector transduced (Vector) MEFs, activated the downstream signalling molecules of MYD88 including the active phosphorylated form of IRAK4^9,10^, as well as activating the phosphorylation of NF-κB p65, a subunit of NF-κB, and phosphorylating IκBα (inhibitor of NF-κB), which induced the activation of NF-κB^9,10,13^. This suggested that the transcriptional activity of NF-κB was induced in these cells. Among these MYD88 constructs, L265P showed a high activation level of MYD88 signals. Indeed, the gene expression of *Nfkbia* (IκBα), a major target of NF-κB-mediated transcription, was induced by the MYD88 constructs (Fig. 1b). Moreover, mRNA expressions of the cytokine *Il6* and chemokines *Mcp1* (monocyte chemoattractant protein-1) and *Cxcl1* (C-X-C motif chemokine ligand 1), targeted genes of NF-κB, were also increased, especially by L265P (Fig. 1b). In accordance with these results, NF-κB p65 and the phosphorylated form of NF-κB p65 were dominant in the nuclei of L265P expressing *p53*^*−/−*^MEFs but not in empty vector expressing cells (Fig. 1c and Fig. S1a). These results indicate that the ectopic expression of MYD88 and its mutants activated NF-κB-mediated transcription in *p53*^*−/−*^MEFs.

Previously, we demonstrated the transcriptional activities of NF-κB were enhanced in *p53*^*−/−*^MEFs, and that activated NF-κB induced the expression of the glucose transporter, type 3 (GLUT3) and the rate of aerobic glycolysis^32^. Moreover, oncogenic RAS-induced cell transformation in *p53*^*−/−*^MEFs was suppressed in the absence of NF-κB p65 expression, and was recovered by GLUT3 expression^32^. This suggested that NF-κB-mediated aerobic glycolysis is important for oncogenesis in the absence of p53 function. As shown in Fig. 1d, the mRNA expressions of glycolysis regulator genes, *Glut1*, hexokinase 2 (*Hk2*), 6-phosphofructo-2-kinase/fructose-2,6-biphosphatase 3 (*Pfkfb3*), and pyruvate dehydrogenase kinase 1 (*Pdk1*) were induced by MYD88 and its mutants. Indeed, enhanced glucose consumption and lactate production, characteristics of enhanced glycolysis, were also observed (Fig. 1e). However, mRNA induction of the NF-κB-target gene *Glut3*^32^ was not induced. Because we previously showed that *Glut1* was not induced by NF-κB in *p53*^*−/−*^MEFs^32^, we analysed the expression of hypoxia-inducible factor-1 (HIF-1), a known transcriptional activator of *Glut1* and the above described glucose regulators^33^. We found that HIF-1α protein, the hypoxia-induced subunit of HIF-1, which is stabilised under hypoxic conditions, was accumulated by MYD88 (Fig. 1f). MYD88-induced HIF-1α had a similar immunoblotting band pattern to that induced by hypoxia (Fig. S1b). These results indicated that HIF-1α protein was induced by MYD88 signals. Indeed, the expression of vascular endothelial growth factor A (*vegfa*), a HIF-1 target gene, was induced in the MYD88 construct-expressing cells (Fig. 1g).

### MYD88-activated NF-κB induces HIF-1α in p53-deficient cells

**Figure 2:**
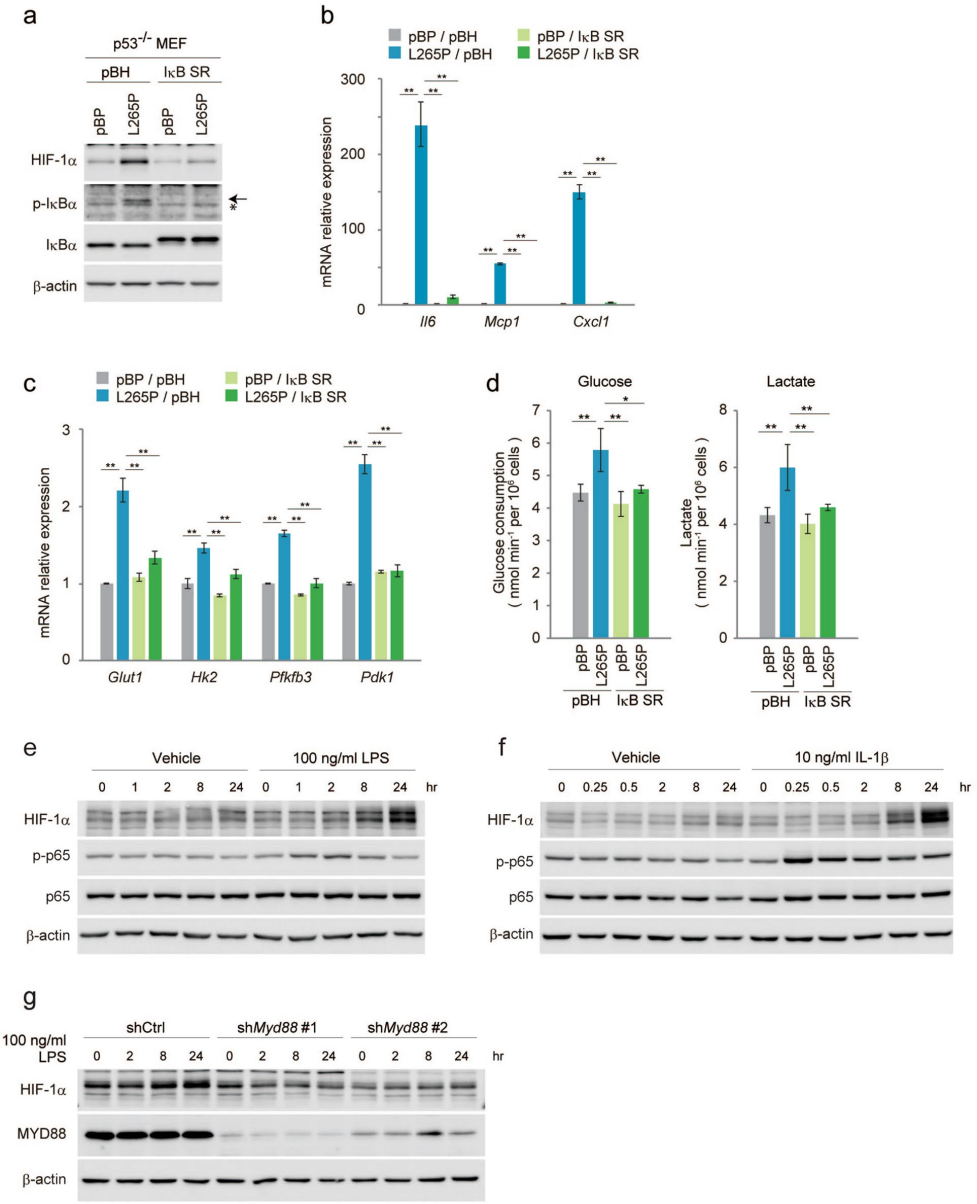
Suppression of NF-κB reduces HIF-1α expression and glucose metabolism in MYD88 L265P-expressing *p53*^*−/−*^MEFs. (**a**–**d**) Suppression of NF-κB using IκB SR in *p53*^*−/−*^MEFs expressing MYD88 L265P. (**a**) Total cell lysates were analysed by immunoblotting. The arrow indicates phosphorylated-IκBα and the asterisk indicates a nonspecific band. (**b**) Gene expression of NF-κB targets measured by qPCR. (**c**) Expression of glucose metabolism-related genes were quantified by qPCR. (**d**) Glucose uptake and lactate production were measured. The quantified results are presented as the mean ± s.d. (n = 4) using one-way ANOVA followed by Scheffe’s F test. *P < 0.05, **P < 0.01. (**e**,**f**) *p53*^*−/−*^MEFs were treated with LPS and IL-1β for the indicated times. (**g**) Endogenous MYD88 expression were reduced by shRNA (#1 and #2), then these cells were stimulated with LPS for the indicated times. (**b**,**c**) The y-axis values are relative fold change for gene transcripts normalised to β-actin. Data represent the mean ± s.d. (n = 3) using one-way ANOVA followed by Scheffe’s F test. *P < 0.05, **P < 0.01.

Next, we analysed whether HIF-1α protein was induced by NF-κB. As shown in Fig. 2a, the protein expression of HIF-1α induced by MYD88 L265P was suppressed by the expression of a dominant-negative inhibitor of NF-κB, IκB-super repressor (IκB SR)^34^. The nuclear localisation of the phosphorylation of NF-κB p65 was inhibited by the expression of IκB SR (Fig. S1a), and the mRNA expressions of NF-κB target genes *Il6*, *Mcp1*, and *Cxcl1* were also inhibited (Fig. 2b, compared to Fig. 1b). Moreover, mRNA expressions of the HIF-1α target genes *Glut1*, *Hk2*, *Pfkfb3,* and *Pdk1*, as well as glucose consumption and lactate production were repressed to nearly the same level as in the controls (Fig. 2c,d compared with Fig. 1d,e). We also knocked-down NF-κB p65 by short hairpin RNA (shRNA) and found that the expressions of HIF-1α protein and glycolysis regulator genes, as well as glucose consumption and lactate production, were decreased (Fig. S2a-d); this was consistent with the results using IκB SR (Fig. 2a–d). This suggested that HIF-1α activation in MYD88 construct-expressing *p53*^*−/−*^MEFs was mediated by NF-κB activation. We further examined HIF-1α expression by NF-κB p65 itself or several stimuli that are known to activate NF-κB^13^. Lipopolysaccharide (LPS), which activates MYD88 through TLR4 and IL-1β that also activates MYD88 through the IL-1 receptor, induced activation of the phosphorylation of NF-κB p65 and the induction of HIF-1α protein (Fig. 2e,f). Moreover, the induction of HIF-1α by LPS was attenuated by the RNA interference-mediated knockdown of *Myd88* (Fig. 2g). These results indicate that MYD88 signals induce the expression of HIF-1α under physiological conditions. However, the forced expression of NF-κB p65 and TNF-α stimulation induced activation of the phosphorylation of NF-κB p65 without the induction of HIF-1α protein (Fig. S3a, b). These results suggest that the NF-κB-mediated induction of HIF-1α protein expression requires NF-κB as well as other MYD88-specific signal(s).

### MYD88-enhanced glycolysis and the expressions of its regulatory genes are mediated by HIF-1

**Figure 3:**
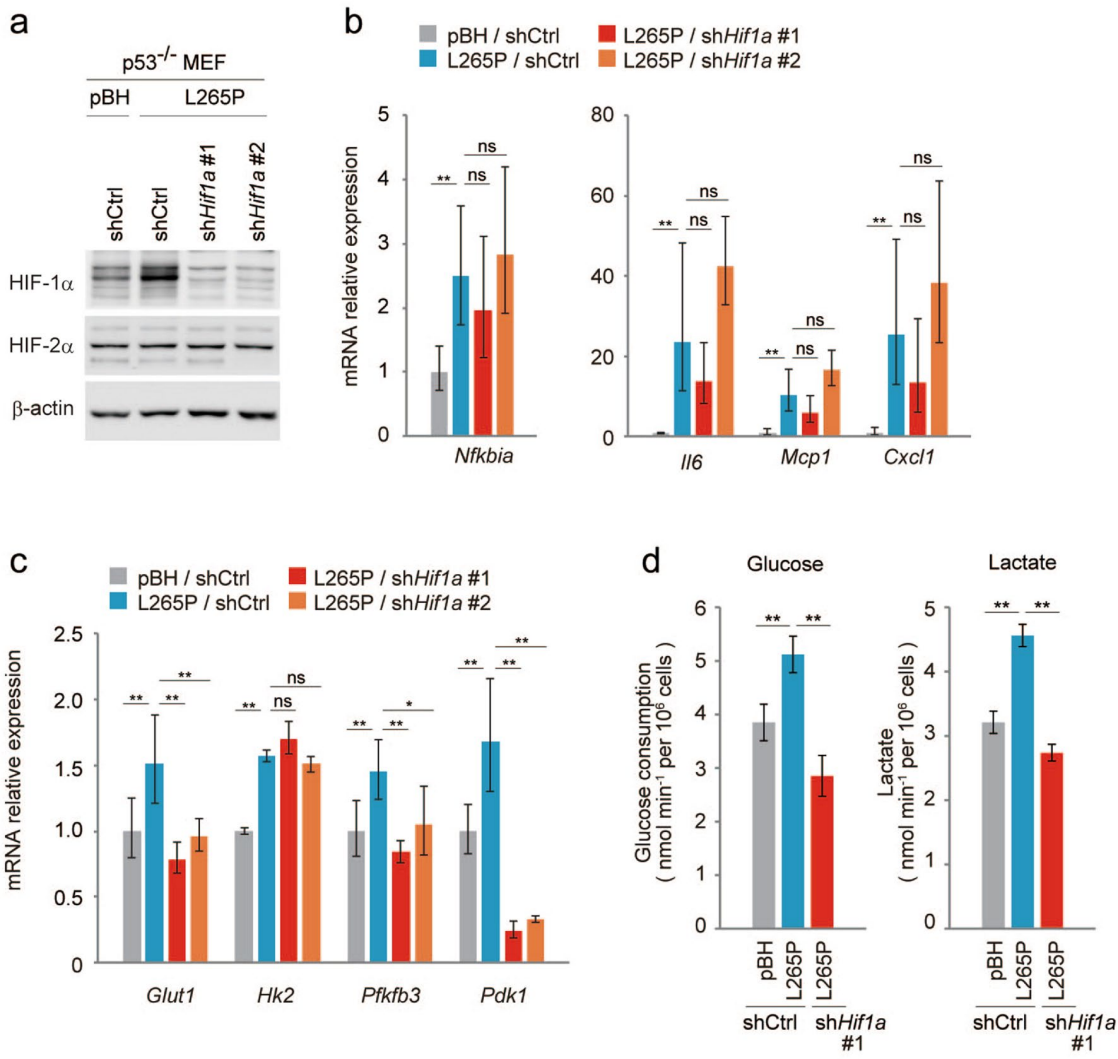
Suppression of HIF-1α does not affect NF-κB activation but reduces glucose metabolism in MYD88 L265P-expressing *p53*^*-/-*^MEFs. Reduced HIF-1α expression by shRNA (#1 and #2) in *p53*^*−/−*^MEFs expressing MYD88 L265P. (**a**) Total cell lysates were analysed by immunoblotting. (**b**) Gene expression of NF-κB targets measured by qPCR. (**c**) Expression of glucose metabolism-related gene mRNAs quantified by qPCR. (**d**) Glucose uptake and lactate production were measured using sh*Hif1a* #1. The quantified results are presented as the mean ± s.d. (n = 4) using one-way ANOVA followed by Scheffe’s F test. *P < 0.05, **P < 0.01. (**b**,**c**) The y-axis values are the relative fold change for gene transcripts normalised to β-actin. Data represent the mean ± s.d. (n = 3) using one-way ANOVA followed by Scheffe’s F test. *P < 0.05, **P < 0.01.

Next, we analysed whether the expressions of glycolysis regulatory genes induced by NF-κB in the MYD88 L265P expressing *p53*^*−/−*^MEFs were mediated by HIF-1α. The expression of HIF-1α was decreased by two different shRNAs, #1 and #2 (Fig. 3a and Fig. S4a), but *Hif1a*-knockdown had little effect on the MYD88-induced expression of NF-κB-target genes (Fig. 3b and Fig. S4b), suggesting HIF-1 does not affect the transcriptional activity of NF-κB. However, expressions of the glycolysis regulator genes, *Glut1*, *Pfkfb3*, and *Pdk1,* but not *Hk2*, were inhibited (Fig. 3c and Fig. S4c). Indeed, *Hif1a*-knockdown in MYD88 L265P-expressing *p53*^*−/−*^MEFs decreased glucose consumption and lactate production (Fig. 3d). In addition, HIF-1α induction by a hypoxia-mimetic agent cobalt chloride (CoCl_2_)^35^ did not affect activation of the phosphorylation of NF-κB p65 (Fig. S3c). These results suggest that MYD88 activates glycolysis through HIF-1α activated by NF-κB.

### HIF-1α protein accumulation by MYD88 is controlled by gene transcription and protein translation

**Figure 4:**
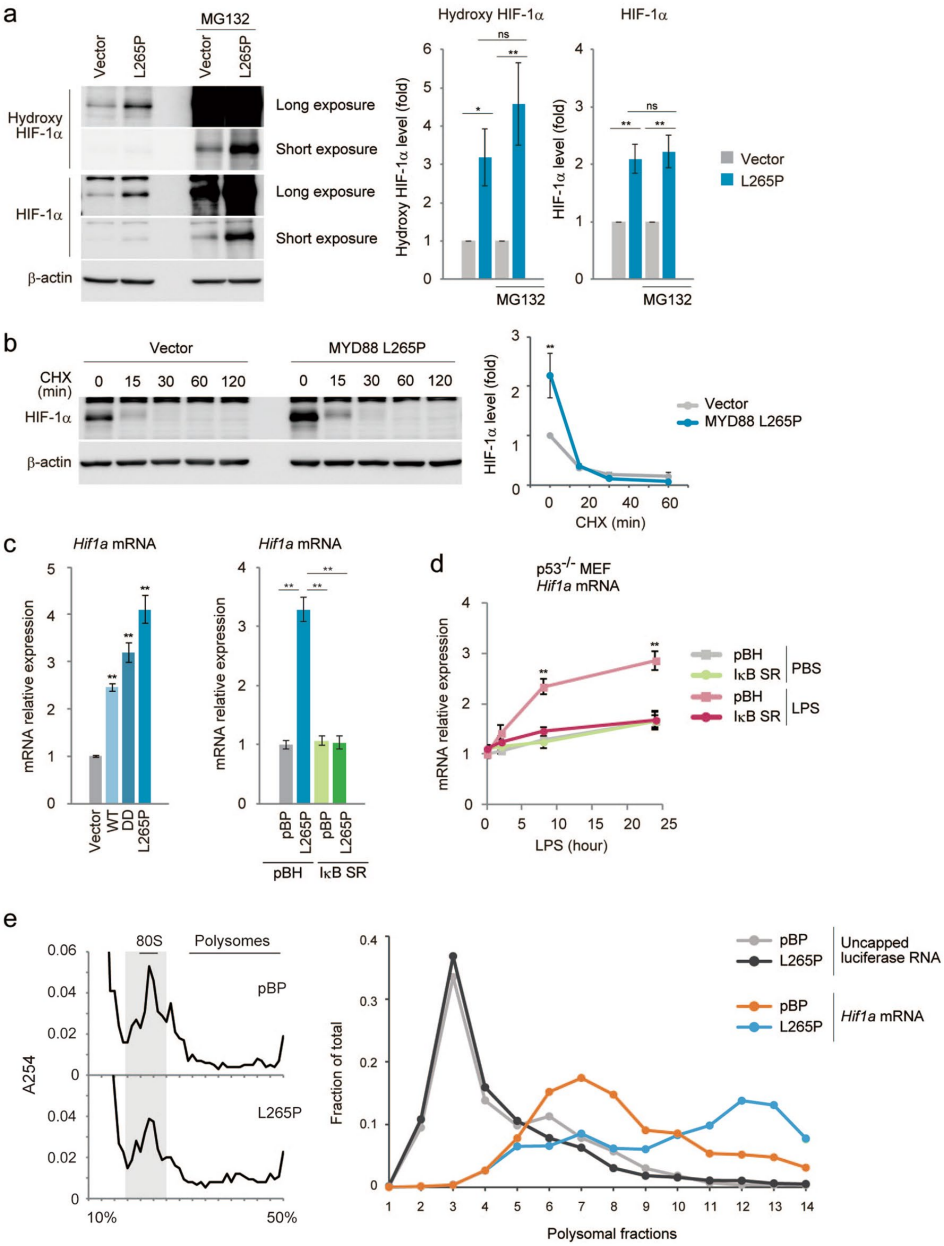
Increased glucose metabolism is mediated by HIF-1α expression regulated at the transcription and translation level in MYD88-expressing *p53*^*−/−*^MEFs. (**a**) *p53*^*−/−*^MEFs expressing vector or MYD88 L265P were treated with 40 μM MG132 for 7 h. Total cell lysates were analysed by immunoblotting (left). Protein expression was quantitated using ImageJ software and the respective protein blots are shown in the graph (right). (**b**) *p53*^*−/−*^MEFs expressing vector or MYD88 L265P were treated with 100 μg/ml cycloheximide (CHX) for the indicated times. Total cell lysates were analysed by immunoblotting (left) and the quantification of HIF-1α signals is shown (right). (**c**) *Hif1a* mRNA expression was quantified in the indicated cells by qPCR. (**d**) *p53*^*−/−*^MEFs were stimulated with 100 ng/ml LPS for 2, 8, and 24 h. Expression of *Hif1a* mRNA was quantified by qPCR. (**e**) Polysomal fractionation was performed for *p53*^*−/−*^MEF expressing vector or MYD88 L265P to detect *Hif1a* mRNA translation efficiency. The sucrose gradient was 10–50% and 15 fractions were collected. The OD254 plot for each polysome profiling experiment (left). The relative distribution of *Hif1a* mRNA associated with each fraction of the gradient was analysed by qPCR (right). The uncapped luciferase RNA was added as an exogenous control, which was not associated with ribosomes and remained in the top fraction. (**c**,**d**) The y-axis values are relative fold change for gene transcripts normalised to β-actin. Data represent the mean ± s.d. (n = 3). (**a**–**d**) The data represent the mean ± s.d. using one-way ANOVA followed by Scheffe’s F test. *P < 0.05, **P < 0.01.

The post-translational regulation of HIF-1α expression has been extensively studied because of its rapid adaptation to hypoxia^33,36^. To determine how HIF-1α expression is regulated in MYD88 L265P expressing *p53*^*−/−*^MEFs, we treated these cells with the proteasome inhibitor MG132. MYD88 L265P expressing cells maintained a high HIF-1α protein expression ratio (Fig. 4a). Moreover, treatment with cycloheximide, a protein translation inhibitor, rapidly reduced the amount of HIF-1α protein in control and MYD88 L265P expressing cells (Fig. 4b). These results suggest that the protein stability of HIF-1α was not affected by MYD88.

**Figure 5:**
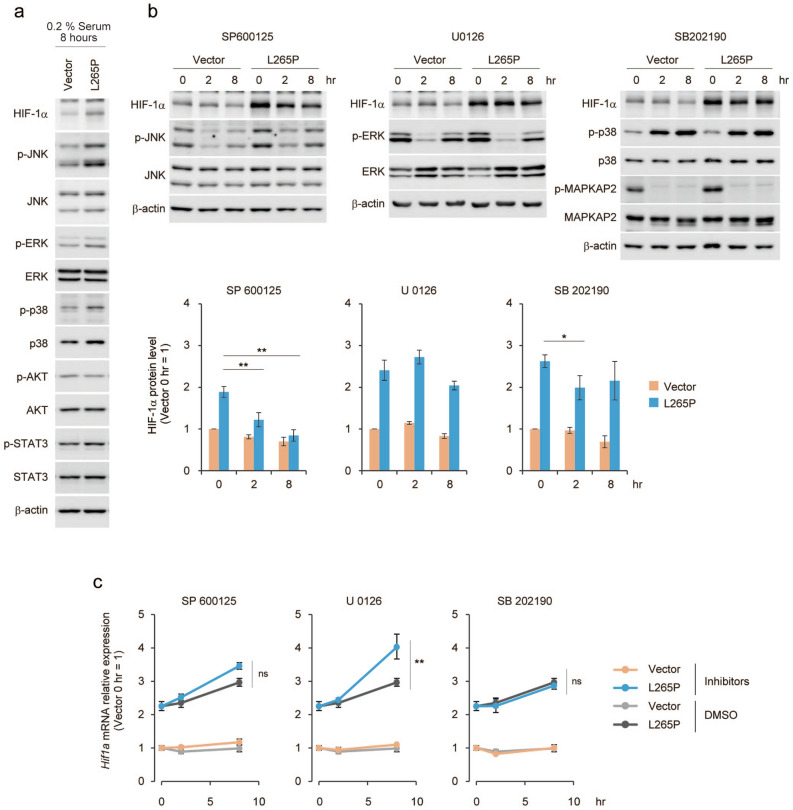
JNK signalling is associated with HIF-1α protein level in MYD88 L265P-expressing *p53*^*−/−*^MEFs. (**a**) Vector and MYD88 L265P-expressing *p53*^*−/−*^MEFs were treated with growth medium containing 0.2% FBS for 8 h. (**b**) Vector and MYD88 L265P-expressing *p53*^*−/−*^MEFs were treated with the indicated MAPK inhibitors. Total cell lysates were analysed by immunoblotting (top). The quantification of HIF-1α signals was measured using ImageJ software and data were normalised to β-actin signals. The data represent untreated controls (vector), which were assigned a value of 1 (bottom). (**c**) *Hif1a* mRNA expression was measured by qPCR. The y-axis values are the relative fold change for gene transcripts. Each value was normalised to β-actin. (**b**,**c**) The data represent the mean ± s.d. using one-way ANOVA followed by Scheffe’s F test. *P < 0.05, **P < 0.01.

A previous study reported that HIF-1α expression was induced by NF-κB^37^, and that LPS induced the mRNA expression of HIF-1α in macrophages^38^. As shown in Fig. 4c and Fig. S2e, MYD88 or its mutants induced the expression of *Hif1a* mRNA, and this induction was not observed by the inhibition of NF-κB activity. Moreover, LPS stimulation induced the expression of *Hif1a* mRNA in *p53*^*−/−*^MEFs and this was reduced by the inhibition of NF-κB activity (Fig. 4d). Moreover, we analysed the translation status of *Hif1a* mRNA, using its association with ribosomes, because the translational activation of specific mRNAs occurred during macrophage activation^39^. *Hif1a* mRNA was present in large polysome fractions^40^ in MYD88 L265P expressing cells compared with control cells (Fig. 4e), suggesting MYD88 signals also activate *Hif1a* mRNA translational efficiency. It was reported that TLR-MYD88 signalling activated the MAP kinase pathway^9,10^, and we found that MYD88 L265P induced activation of the phosphorylation of the MAP kinase pathway factors, JNK, ERK, and p38 (Fig. 5a). Therefore, we examined whether the MAP kinase pathway was involved in HIF-1α expression. Inhibitors of JNK, but not ERK or p38, significantly reduced HIF-1α protein expression over time (Fig. 5b). Moreover, although the ERK inhibitor increased *Hif1a* mRNA expression by an unidentified mechanism, it was not inhibited by these inhibitors (Fig. 5c). Therefore, MYD88 induced-JNK signalling might affect HIF-1α protein expression levels. However, a JNK inhibitor did not affect the pattern of *Hif1a* mRNA present in the large polysome fraction (Fig. S5a) and HIF-1α protein stability (Fig. S5b). A previous study reported that inhibitors of protein translation initiation induced ribosome run-off whereas inhibitors of elongation and termination stabilized polysomes^41^. Therefore, JNK might regulate HIF-1α protein elongation and termination. Further analyses are required to clarify the mechanism underlying the effect of JNK.

### MYD88 activation promotes the generation of TIC-like cells

**Figure 6:**
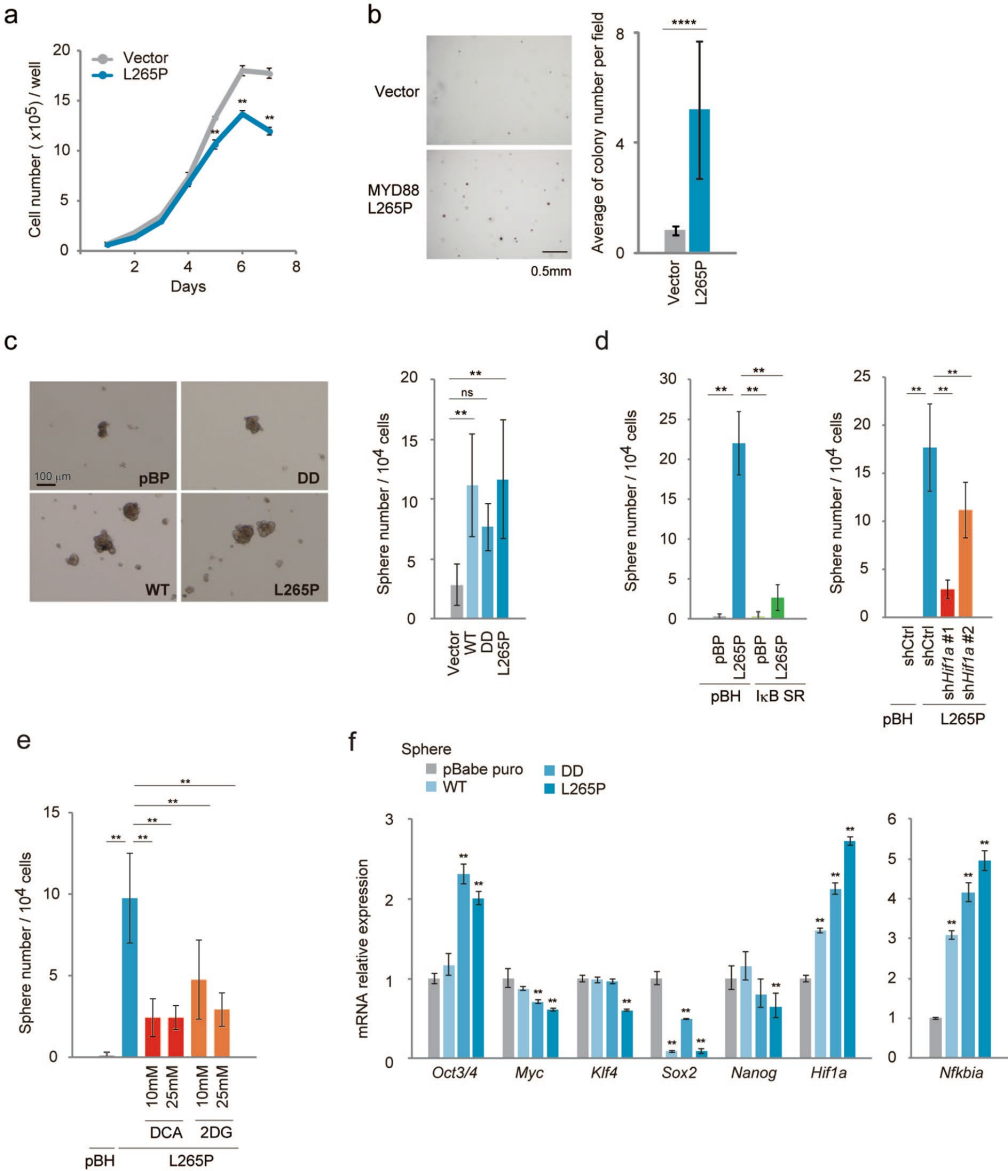
Spheres from *p53*^*−/−*^MEFs expressing activated MYD88 constructs express *Oct3/4* genes. (**a**) A cell growth assay was performed using the indicated cells. Cell numbers were counted every day between day 0 and day 7 (n = 3). (**b**) Colony formation assay using stably expressing vector and MYD88 L265P constructs in *p53*^*−/−*^ MEFs. Cells (25 × 10^3^ per well) were seeded in soft agar and incubated for 4 weeks. Colonies with a diameter > 35 μm were counted. Left panel: representative images of the colony. Scale bars, 0.5 mm. Right panel: graphic represents three independent experiments. A two-tailed *t*-test was used for statistical analysis. The mean ± s.d. is shown. ****P < 0.0001. (**c**) Sphere formation assays were performed using stably expressing vector and MYD88 constructs in *p53*^*−/−*^ MEFs. Left panel: representative images of spheres. Scale bars, 100 μm. Right panel: the mean number of spheres per 10^4^ cells from three independent experiments. (**d**) Sphere formation assays were performed: (left) suppression of NF-κB by IκB SR; (right) reduced HIF-1α expression by sh*Hif1a* #1 and #2, in *p53*^−/−^ MEFs expressing MYD88 L265P. (**e**) Sphere formation assays. The glucose metabolism pathway was inhibited using a PDK inhibitor dichloroacetate (DCA) and glycolysis inhibitor 2-deoxy-D-glucose (2DG), in *p53*^−/−^ MEFs expressing MYD88 L265P. DCA and 2DG were used at the indicated concentrations. (**c**–**e**) Numbers of spheres with a diameter > 100 μm were counted between days 6 and 9. The quantified results are presented as the mean ± s.d. using one-way ANOVA followed by Scheffe’s F test. *P < 0.05, **P < 0.01. (**f**) Expression of reprogramming-related genes from spheres was quantified by qPCR. The y-axis values are the relative fold change for gene transcripts normalised to β-actin. Data represent the mean ± s.d. (n = 3) using one-way ANOVA followed by Scheffe’s F test. *P < 0.05, **P < 0.01.

Next, we assessed the relevance of MYD88 activation in tumour development. MYD88 L265P expressing *p53*^*−/−*^MEFs had a slightly lower proliferation rate than the control *p53*^*−/−*^MEFs (Fig. 6a). Consistent with this, the expression levels of cell cycle regulator proteins cyclin D, E, A, and B were not significantly different (Fig. S6). In contrast, a soft agar colony formation assay demonstrated that MYD88 L265P promoted anchorage-independent growth in vitro, which is characteristic of TICs^42^ (Fig. 6b). We also performed a sphere formation assay, a useful tool to assess TIC populations, using *p53*^*−/−*^MEFs expressing MYD88 constructs, and found that these cells formed significant numbers of spheres (Fig. 6c). Moreover, the inhibition of NF-κB by IκB SR and HIF-1α knockdown inhibited sphere numbers (Fig. 6d), suggesting that the MYD88-activated NF-κB-HIF-1α pathway is important for TIC generation. However, sh*Hif1a* #2-expressing cells formed relatively high numbers of sphere forming cells compared with shHif1a #1-expressing cells (Fig. 6d). We also found that the mRNA expression levels of HIF-1 target genes, *Glut1, Pfkfb3,* and *Pdk1* were significantly higher in sh*Hif1a* #2-expressing sphere cells than in sh*Hif1a* #1-expressing sphere cells (Fig. S7). These results were consistent with data showing that the inhibition of *Hif1a* mRNA expression by sh*Hif1a* #2 cells was relatively weak compared with sh*Hif1a* #1 cells (Fig. S7). However, the expression levels of HIF-1 target genes in L265P/sh*Hif1a* #1 and #2 cells were lower than those in control cells not expressing L265P. These results suggest that signal pathway(s) other than HIF-1 were induced by L265P in cooperation with HIF-1 to generate TICs.

**Figure 7:**
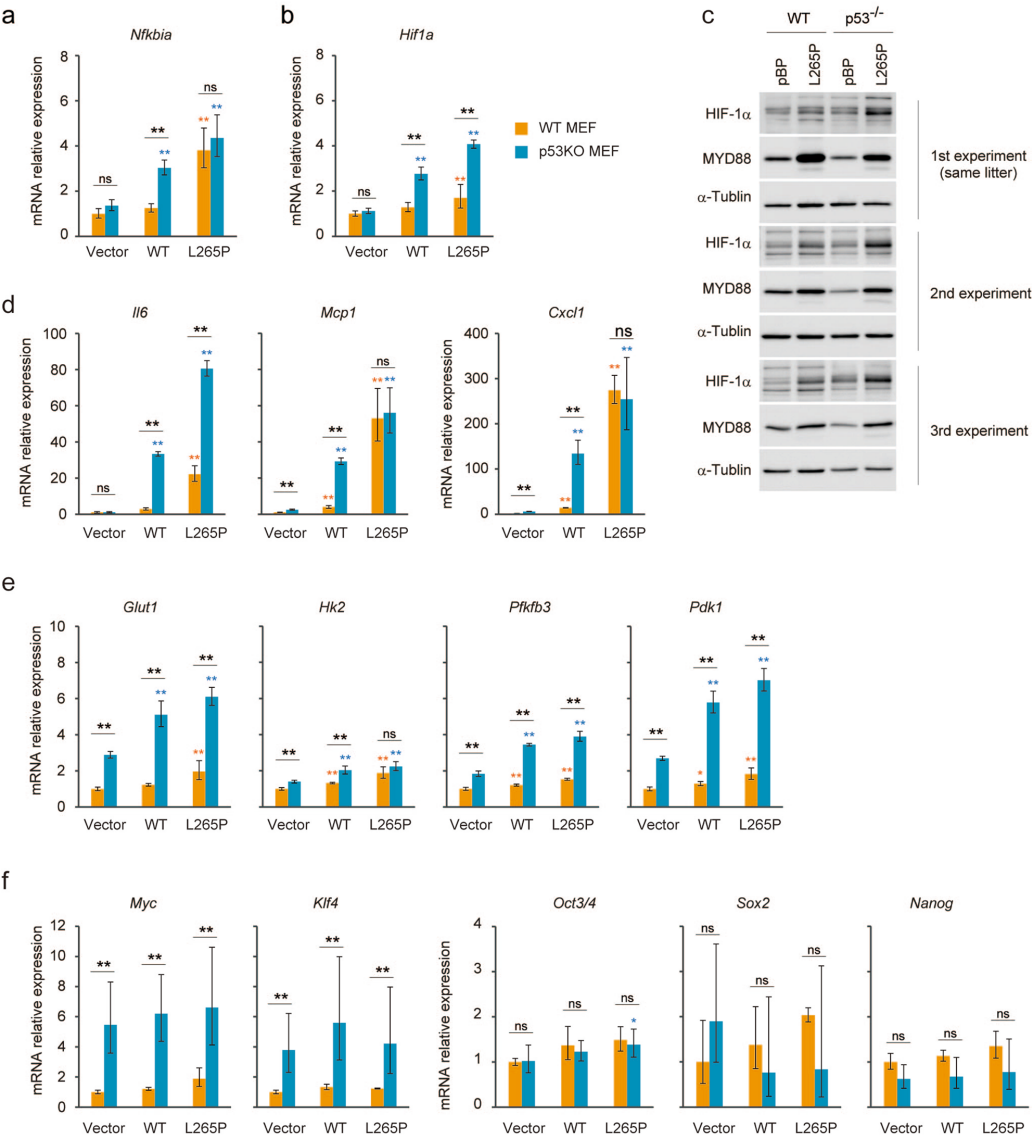
Deficiency of p53 promotes increased HIF-1 signalling. (**a**,**b**,**d**–**f**) The indicated MYD88 constructs were introduced by retroviral infection to wild type and *p53*^*−/−*^MEFs. Expressions of the indicated mRNAs were measured by qPCR. The y-axis values are relative fold change for gene transcripts normalised to β-actin. The data represent the mean ± s.d. (n = 3) using one-way ANOVA followed by Scheffe’s F test. *P < 0.05, **P < 0.01. The black asterisks show significance between WT and *p53*^*−/−*^MEFs. The orange asterisks show significance between indicated cells and vector introduced WT MEFs. The blue asterisks show significance between indicated cells and vector introduced *p53*^*−/−*^MEFs. (**c**) WT and *p53*^*−/−*^MEFs from the same litter or same background were used for this experiment. Total cell lysates were analysed by immunoblotting.

In addition to enhanced glycolysis, it was shown that the HIF-1-mediated expression of PDK1 is required for cellular adaptation to hypoxia^43^. As shown in Fig. 6e, the glycolysis inhibitor 2-deoxy-D-glucose (2-DG) and PDK1-4 inhibitor dichloroacetate (DCA) inhibited MYD88 L265P-induced sphere cell generation, suggesting that HIF-1-induced metabolic reprogramming is involved in the generation of TICs. Furthermore, the mRNA expressions of reprogramming factors in MYD88 L265P-expressing spheres showed that *Oct3/4* mRNA expression was higher than in spheres from control *p53*^*−/−*^MEFs (Fig. 6f), but marked induction was not observed in cells from a conventional adherent culture (Fig. 7f and Fig. S8a). However, high expressions of other reprogramming factors *Myc*, *Klf4,* and *Nanog*^44^ were observed in spheres compared with conventional adherent cells (Fig. S8a), but this was not enhanced by MYD88 constructs in the spheres (Fig. 6f), suggesting OCT3/4 is an effector molecule of the MYD88-HIF-1-mediated generation of cancer stem cell-like cells. Moreover, *Nfkbia* mRNA, a parameter of NF-κB activation, and *Hif1a* mRNA expressions remained high in MYD88 construct-expressing spheres (Fig. 6f) compared with adherent cells (Fig. S8b), suggesting that NF-κB and HIF-1 activities were enhanced in the spheres. These results suggest that activation of the NF-κB-HIF-1 pathway enhanced the stepwise stochastic process of reprogramming^44^ with a relatively high expression of OCT3/4, a major regulator of cell pluripotency^45^.

### Role of p53-deficiency in the generation of TIC-like cells induced by MYD88 signalling

Several studies have shown that p53 inhibited the reprogramming of somatic cells to stem cells^46^, and that inactivation of p53 increased the efficiency of iPSC generation induced by MYC, KLF4, SOX2, and OCT3/4^25,27–29^. We previously found that the transcriptional activity of NF-κB was suppressed by p53 and that oncogenic RAS-induced tumourigenic cell transformation in *p53*^*−/−*^MEFs was dependent on NF-κB p65^32,47^, suggesting a role of NF-κB in the induction of TIC-like properties. Furthermore, MYD88 WT-induced *Nfkbia* mRNA expression was suppressed in p53-expressing WT MEFs (Fig. 7a). The transcriptional activity of NF-κB was similarly induced by MYD88 L265P in the presence or absence of *p53* indicating the suppression of NF-κB by p53 itself is not a major function of p53-mediated reprogramming barrier against MYD88. In contrast, the NF-κB-dependent induction of *Hif1a* mRNA and protein expressions were markedly suppressed by the presence of p53 (Fig. 7b,c), suggesting the existence of a selective inhibitory mechanism of NF-κB-target genes by p53 in response to MYD88 signals. This selective inhibition was also supported by data showing that among the MYD88-inducible NF-κB-target genes *Il6*, *Mcp1,* and *Cxcl1* (Fig. 2b), only the induction of *Il6* mRNA by MYD88 L265P was suppressed by the presence of p53 (Fig. 7d).

Regarding the role of NF-κB in *p53*^*−/−*^MEFs, we previously found that enhanced glycolysis by NF-κB was critical for tumourigenic transformation^32^. We also found that the O-linked β-N-acetyl glucosamine (O-GlcNAc) modification of proteins (O-GlcNAcylation), which is usually increased by enhanced glycolysis, was required for the generation and maintenance of TICs of colon and lung cancer cells^48^. Therefore, enhanced glycolysis induced by the activation of the MYD88 pathway and by the absence of p53-mediated glycolysis inhibition might be involved in the generation of TIC-like cells. Indeed, the MYD88-NF-κB-HIF-1 pathway-induced expressions of HIF-1-regulated glycolysis-related genes, *Glut1*, *Pfkfb3,* and *Pdk1*, but not *Hk2* (Fig. 3c) were markedly attenuated in the presence of p53 (Fig. 7e). In addition, the mRNA expressions of reprogramming factors were not induced by MYD88 constructs under conventional cell culture conditions; however, the mRNA expression levels of the oncogenic reprogramming factor genes, *Klf4* and *Myc,* which may confer increased proliferative capacity on potential iPS cells^44^, were suppressed in p53-expressing cells (Fig. 7f). These results suggest that the p53-mediated attenuation of HIF-1 activity induced by MYD88 signals (Fig. 7e) is involved in the p53-mediated barrier function against the generation of TIC-like cells.

### MYD88 signal activating cells develop tumours in nude mice via NF-κB and HIF-1α

**Figure 8:**
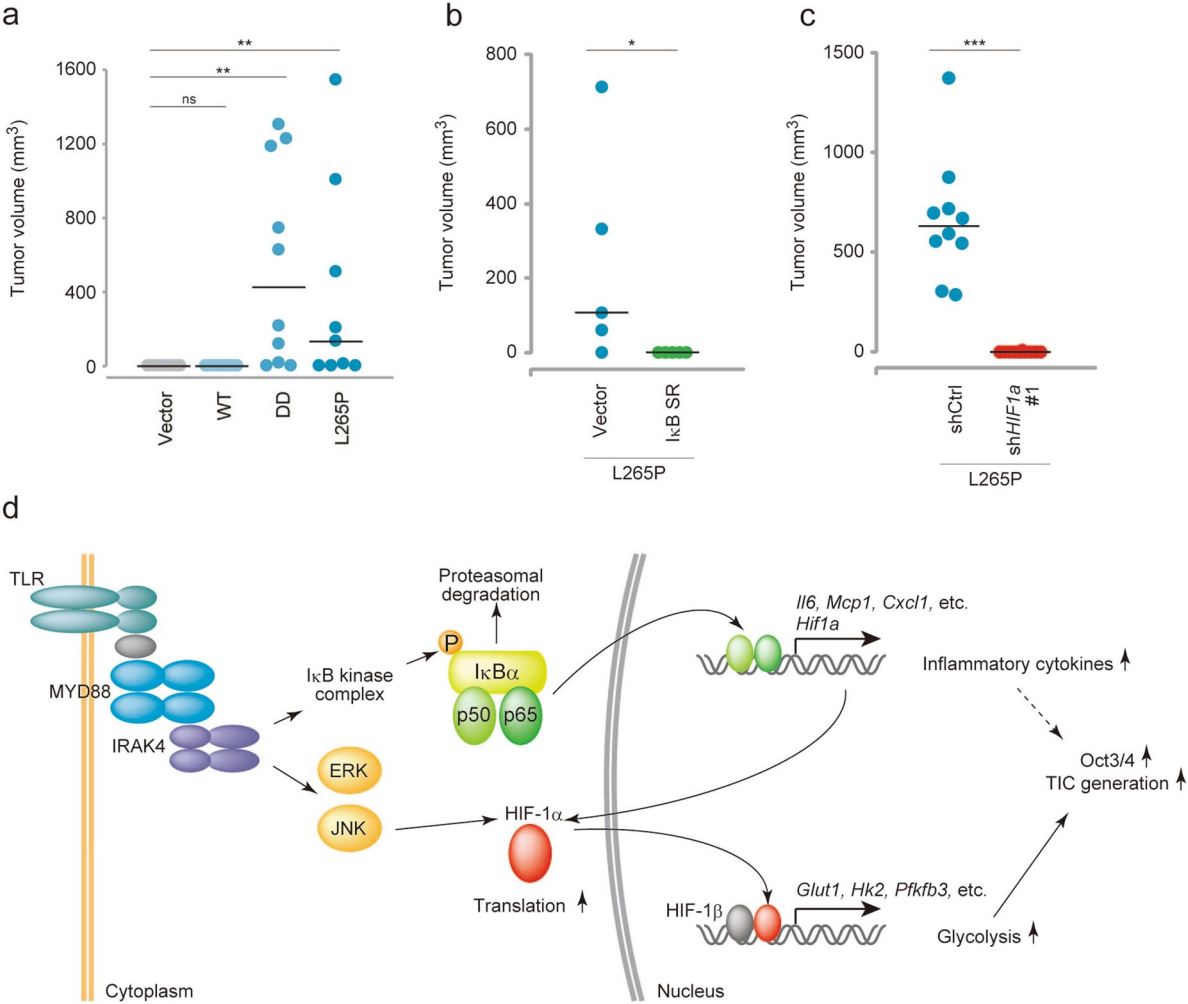
MYD88 L265P expressing *p53*^*−/−*^MEFs form tumours via the NF-κB-HIF-1α axis. (**a**–**c**) Tumourigenesis experiments in vivo. Tumour sizes were monitored weekly. (**a**) The indicated gene-expressing *p53*^*−/−*^MEFs (2.5 × 10^6^ cells) were subcutaneously injected into 7-week-old nude mice (n = 10 per group, except L265P, n = 9). One mouse in the L265P group formed a tumour and was eliminated from this statistical analysis because of unknown death. (**b**) Vector or IκBSR introduced MYD88 L265P-expressing *p53*^*−/−*^MEFs (1.6 × 10^6^ cells) were subcutaneously injected into 7-week-old nude mice (n = 6 per group). (**c**) shCtrl or sh*Hif1a* introduced MYD88 L265P-expressing *p53*^*−/−*^MEFs (2.5 × 10^6^ cells) were subcutaneously injected into 7-week-old nude mice (n = 10 per group). (**d**) A graphical depiction of the mechanism of MYD88-induced TIC generation from *p53*^*−/−*^MEFs. (**a**–**c**) For the statistical analysis, the Kruskal–Wallis test followed by the Steel–Dwass test as a post-hoc test were used for (**a**) and the Mann–Whitney *U*-test was used for (**b**,**c**) *P < 0.05, **P < 0.01, ***P < 0.001.

Finally, we analysed whether MYD88 signals induced TIC generation in *p53*^*−/−*^MEFs using a tumour xenograft assay in nude mice. Cells were inoculated subcutaneously into mice and tumour growth was monitored each week. The expression of constitutively active MYD88 mutants, DD or L265P, but not WT MYD88, resulted in the efficient induction of tumour growth (Fig. 8a). Suppression of NF-κB activity by IκB SR significantly inhibited tumour growth (Fig. 8b). Furthermore, *Hif1a* knockdown also inhibited tumour growth (Fig. 8c), even though the cell growth of sh*Hif1a* with MYD88 L265P was faster than that of MYD88 L265P expressing *p53*^*−/−*^MEFs (Fig. S4d). These results show that activated MYD88 signalling promoted TIC generation via the NF-κB-HIF-1α activation cascade (Fig. 8d).

## Discussion

Tumour-promoting inflammation is a hallmark of cancer, and chronic inflammation increases the risk of cancers^49^. Inflammatory cells, such as neutrophils, macrophages, and lymphocytes, migrate to the inflamed tissues and secrete inflammatory cytokines and chemokines. These cytokines and chemokines induce the recruitment of mesenchymal stem cells that regulate the local inflammatory microenvironment and the repair activities of tissue stem cells^50,51^. Moreover, growth factors^52^ and inflammatory cytokines^53–55^ enhance the energy metabolism in target cells. Therefore, such an inflammatory microenvironment might contribute to oncogenesis by supplying bioactive molecules including growth, survival, proangiogenic, and invasion- and metastasis-inducing factors that lead to the induction of cancer-facilitating programs^49^. In this context, it was demonstrated that epithelial barrier defects resulted in the invasion of microbes that triggered tumour-elicited inflammation and the production of IL-23 and IL-17, which enhanced tumour growth in APC mice^18^. This result suggests that inflammation induced by microbes promoted oncogenesis through inflammatory cytokines, and this crosstalk between cancers, immune cells, and microorganisms has been extensively analysed^12^. In the present study, we found that MYD88 signals induced TIC generation through NF-κB-HIF-1α. Although this mechanism may not be the only cause of inflammation-induced cancer, this result indicates that MYD88 has a cell-intrinsic tumour-initiating activity and that the constitutive infection of microbes is sufficient for oncogenesis in the absence of immune cells.

The current results indicate that the activation of NF-κB and HIF-1α is important for TIC generation. It was previously demonstrated that NF-κB promoted TIC development and maintenance through the induction of the epithelial-to-mesenchymal transition (EMT) inducers, SLUG, TWIST1, and SNAIL^13^. Moreover, we previously demonstrated that the transcriptional activity of NF-κB as well as glycolysis was enhanced in *p53*^*−/−*^MEFs, and that oncogenic RAS-induced cell transformation and enhanced aerobic glycolysis in *p53*^*−/−*^MEFs were dependent on NF-κB^32^. These results suggest that NF-κB-mediated enhanced aerobic glycolysis is also important for TIC generation. Moreover, in *p53*^*−/−*^MEFs, O-GlcNAcylation was enhanced by NF-κB-mediated enhanced glycolysis^56^. Furthermore, O-GlcNAcylation regulated the reprogramming of somatic cells to iPSCs and their pluripotency by modification of the core reprogramming factors OCT3/4 and SOX2^57^. Related to this, we found that IL-8-induced O-GlcNAc modification was required for the generation and maintenance of TICs of colon and lung cancer cells^48^. Although several mechanisms might be involved in TIC regulation by NF-κB, the current results indicate that NF-κB-induced HIF-1α activation is essential for TIC generation in MYD88 signal-activated *p53*^*−/−*^MEFs.

HIF-1 regulates the expression of genes that contribute to angiogenesis, metabolic reprogramming, extracellular matrix remodelling, EMT, motility, invasion, metastasis, cancer stem cell maintenance, immune evasion, and resistance to chemotherapy and radiation therapy^58^. During TIC generation and maintenance, HIF-1α regulated the expression of the Hippo pathway effector TAZ, and the stem cell markers CD44 and OCT3/4^59^. Indeed, the expression of *Oct3/4* was decreased in colorectal cancer cells in response to HIF-1α knockdown^60^. In the current study, we found that the mRNA expression of *Oct3/4* was enhanced in sphere-forming cells; however, this transcriptional activation was not directly caused by HIF-1α. As in iPSCs, stem cell-reprogramming occurred through several steps^44^, and our results suggested that HIF-1α might induce a set of factors that activate the reprogramming step. Furthermore, several studies showed that the oncogenic signal pathways ERK/MAPK, JAK/STAT, and PI3K/Akt/mTOR increased the transcription and translation of HIF-1α in cancer^61,62^. Our current results also showed that NF-κB-mediated transcriptional activation and enhanced translation of HIF-1α protein induced the activation of HIF-1α in response to MYD88 signals. However, the mechanism involved in the induction of HIF-1α protein downstream of the JNK signal is unclear but might involve enhanced translation. Therefore, to investigate the mechanism of inflammation-induced TIC generation and treatment of cancer induced by chronic inflammation, it is important to determine the molecular mechanism of HIF-1α induction in response to MYD88 signals in more detail.

p53 was demonstrated to inhibit inflammatory responses, and its functional loss caused excessive inflammatory reactions^63^. This suggested that p53 suppresses inflammation-induced tumour development by limiting the inflammatory response. Moreover, p53 functions as a barrier against stem cell reprogramming^25–29^. Then, what mechanism(s) operate in the p53-mediated suppression of TIC generation in response to inflammatory signals? It was reported that the overexpression of reprogramming factors triggered premature senescence mediated by p53^64^. Therefore, the induction of senescence by oncogenes is a tumour-suppressive mechanism of p53^65^. Moreover, p53 repressed the expression of many genes encoding key regulators of embryonic stem cells, including the reprogramming factors *Oct3/4*, *Nanog*, and *Sox2*^66^. Furthermore, recent findings suggested that oncogenic mutations under an inflammatory microenvironment promoted cancer development through chromatin remodelling caused by epigenetic plasticity^63^. Therefore, the loss of p53 function might induce reprogramming factors in response to tumour-promoting inflammatory signals such as MYD88. Another possible mechanism is that the metabolic regulation of p53 suppresses TIC generation^67^. In the present study, we found that the expressions of *Hif1a* and HIF-1-regulated genes involved in glucose uptake and metabolism were upregulated in *p53*^*−/−*^MEFs in response to MYD88 signals. Compared with the present results, a previous study reported that LPS effectively induced *Hif1a* mRNA and protein in p53-expressing mouse bone marrow-derived macrophages^68^. We analysed MEFs and *p53*^*−/−*^MEFs from three age-matched wild-type C57BL/6 mice on the same genetic background by performing additional backcrosses and obtained the same result (Fig. 7c). Moreover, the induction of HIF-1 target gene expressions was lower in wild-type MEFs compared with *p53*^*−/−*^MEFs, indicating p53 suppresses HIF-1α expression and activity in MEFs. Therefore, our results strongly suggest that MYD88-activated HIF-1 induces TIC generation in some cell types through enhanced glucose metabolism in the absence of p53, in cooperation with its ability to induce reprogramming factors^59^.

In conclusion, the current study clearly showed that MYD88 signals induced TIC generation though the NF-κB-HIF-1α activation cascade and that activation of the JNK pathway was also involved in HIF-1α activation. Although our experiment using mouse cells might not reflect all the processes of human inflammation-induced cancer, the identification of the basic regulatory mechanism of the MYD88-NF-κB-HIF-1α activation pathway and its involvement in TIC production is important. To develop preventative and therapeutic methods for inflammation-induced cancer, further analyses of the detailed molecular regulatory mechanism of MYD88-induced TIC generation are required.

## Methods

### Cell culture and reagents

MEFs were prepared as described previously^32^. The HEK293T cell line and wild-type and *p53*^*−/−*^MEFs were cultured in Dulbecco’s modified Eagle’s medium supplemented with 10% fetal bovine serum (FBS). The following reagents were used for treating cells: cycloheximide (Cat# 06741-04; Nacalai Tesque, Inc., Kyoto, Japan), MG132 (Cat# 474790; Merck KGaA, Darmstadt, Germany), lipopolysaccharide (Cat# L2637; Merck KGaA), TNF-α (Cat# 300-01A; PeproTech, Inc., Rocky Hill, NJ, USA), IL-1β (Cat# 200-01B; PeproTech, Inc.), dichloroacetate (Cat# 347795; Merck KGaA), and 2-deoxy-D-glucose (Cat# D8375; Merck KGaA).

### Retroviral vectors and infection

The full-length and death domain of mouse *Myd88* was amplified from mouse cDNA using PrimeSTAR GXL DNA polymerase (Takara, Shiga, Japan), cloned into pT7Blue Vector (Merck KGaA), and then a l252p mutation (equivalent site to L265P in human MYD88) was introduced by site-direct mutagenesis using the sense primer 5′-ggtgtccaacagaagcgacctattcctattaaatacaaggc-3′ and antisense primer 5′-gccttgtatttaataggaataggtcgcttctgttggacacc-3′. These *Myd88* constructions were added into pBabe-puro and -hygro vectors. IκB SR cloned into the pBabe-hygro vector was kindly provided by Takashi Fujita, Kyoto University, Japan. The shRNA-targeted sequences against mouse *Hif1a* were synthesised using the following: 5′-GAATCAAGAGGTTGCATTA-3′ (#1) and 5′-GGAAGGTATGTGGCATTTA-3′ (#2). Oligo pairs were annealed and subcloned into the polylinker region of the pSUPER.retro.puro vector. The shRNA-targeted sequence against mouse p65 was described previously^32^. Retroviral infection was performed as described previously^32^. The shRNA-targeted sequences against mouse *Myd88* were synthesised using the following: 5′-GCCAGCGAGCTAATTGAGAAA-3′ (#1) and 5′-CCTTTCACGTTCTCTACCATA-3′ (#2). Oligo pairs were annealed and subcloned into the third-generation lentivirus pLKO.1 puro vector. Infected cells were selected using puromycin (2 μg/ml) and hygromycin (200 μg/ml).

### Immunoblotting

Protein extracts were resolved by SDS-PAGE and transferred to nylon membranes (Immobilon-P; Merck KGaA) using standard techniques. Primary antibodies used for immunoblotting were as follows: MYD88 (sc-11356; Santa Cruz Biotechnology, Inc., Dallas, TX, USA) for the detection of the N-terminal of MYD88, MYD88 (#4283; Cell Signaling Technology, Inc., Danvers, MA, USA), phospho-IRAK4 (#11927; Cell Signaling Technology, Inc.), IRAK4 (#4363; Cell Signaling Technology, Inc.), phospho-p65 (#3033; Cell Signaling Technology, Inc.), p65 (sc-372; Santa Cruz Biotechnology Inc.), phospho-IκBα (#9246; Cell Signaling Technology, Inc.), IκBα (sc-371; Santa Cruz Biotechnology, Inc.), HIF-1α (NB100-479; Novus Biologicals, Centennial, CO, USA), HIF-2α (NB100-122; Novus Biologicals), hydroxy-HIF-1α (#3434; Cell Signaling Technology, Inc.), phosphor-JNK (#9251; Cell Signaling Technology, Inc.), JNK (#9252; Cell Signaling Technology, Inc.), phosphor-ERK (#9101; Cell Signaling Technology, Inc.), ERK (#9102; Cell Signaling Technology, Inc.), phosphor-p38 (#9211; Cell Signaling Technology, Inc.), p38 (#9212; Cell Signaling Technology, Inc.), phosphor-MAPKAP2 (#3316; Cell Signaling Technology, Inc.), and MAPKAP2 (#12155; Cell Signaling Technology, Inc.).

### Immunofluorescence staining

MEFs were seeded on glass coverslips, washed with PBS and fixed in 4% paraformaldehyde. Following fixation, the cells were washed with PBS, permeabilised for 10 min in 0.2% Triton X-100/PBS, blocked with 10% goat serum in PBST (0.1% Tween 20/PBS) for 1 h, and then incubated with a primary antibody, p65 (sc-372; Santa Cruz Biotechnology, Inc.) or phosphor-p65 (#3033; Cell Signaling Technology, Inc.) at 4 °C overnight. After three times wash with PBST, the cells were incubated for 1 h with secondary antibody, Alexa Fluor 488-conjugated anti-rabbit IgG (A11070; Thermo Fisher Scientific Inc., Waltham, MA, USA), and then washed three times with PBST. DNA was counterstained using DAPI (D9542; Merck KGaA). Coverslips were mounted onto slides using ProLong Gold Antifade Mountant (P36934; Thermo Fisher Scientific Inc.). All images were taken by a confocal microscope (FV1200; Olympus Corporation, Tokyo, Japan).

### Real-time quantitative PCR

Total RNA from each MEFs was extracted using NucleoSpin RNA (Macherey–Nagel GmbH & Co. KG, Düren, Germany), and was reverse transcribed with PrimeScript RT reagent Kit (Takara). Real-time quantitative PCR was performed using TaqMan Gene Expression Master Mix and TaqMan probe by StepOne Real-Time PCR System (Thermo Fisher Scientific Inc.)^32^. The samples were run in triplicate and normalised to *Actb* using a ∆∆ cycle threshold-based algorithm, to serve arbitrary units representing relative expression levels. ∆∆ CT values were used for the statistical analysis using one-way ANOVA followed by Scheffe’s F test. All data are presented as the mean ± s.d. of at least three independent experiments.

### Cell proliferation assay

Indicated cells were seeded into six-well plates with a density of 4 × 10^4^ cells/well in triplicate. Every day for a week, the cells were harvested and counted by Vi-CELL XR (Beckman Coulter, Inc. Brea, CA, USA) as previously described^45^.

### Colony formation assay

Cells were trypsinised and filtered through a 40-μm cell strainer. A layer of 0.75% (wt/vol) agarose in normal medium was prepared in six-well plates and a layer of 0.36% agarose containing 25 × 10^3^ infected cells was poured over the first layer. After 4 weeks, colonies greater than 35 μm in diameter were counted under a microscope in five fields per well. Three independent experiments were performed in triplicate.

### Sphere formation assay

Cells were trypsinised and filtered through a 40-μm cell strainer. Single cells were suspended in DMEM/F12 with 20 ng/ml EGF (Fujifilm Wako Pure Chemical Corporation, Osaka, Japan) and 10 ng/ml bFGF (Fujifilm Wako Pure Chemical Corporation) without serum at a density of 1 × 10^4^ cells in a six-well ultra-low attachment plate. After 6–9 days, spheres were collected for RNA extraction, and the sphere number was counted.

### Measurements of glucose consumption and lactate production

These methods were modified from previous work^32^. The cells were seeded in culture dishes (four dishes for each cell type) and the medium was changed the next day. Cells were incubated for 18 h and the culture medium was collected for the measurement of glucose and lactate concentrations. Glucose levels were determined using a Glucose (GO) assay kit (Merck KGaA). Glucose consumption was determined from the difference in glucose concentration compared with control dishes without cells. Lactate levels were determined using F-kit L-lactate (J. K. International, Inc., Tokyo, Japan). After culture medium was collected, cells were trypsinised and counted. Three or more independent experiments were performed in quadruplicate.

### Polysome fractionation

The polysomal fraction was isolated from cells as previously described^40^. Briefly, the cells were washed twice with PBS containing 100 μg/ml cycloheximide (CHX) and lysed with lysis buffer. Exogenous uncapped luciferase mRNA was added to a final concentration of 100 ng/ml. The lysate was fractionated using a sucrose gradient (10–50% sucrose) at 160,000 × *g* for 2.5 h at 4 °C using a Beckman Coulter SW41Ti rotor. Forty-five fractions were collected manually and the optical density was measured at 254 nm using a NanoDrop 2000. Three fractions were combined and fifteen fractions were used for further analyses. RNA from each sample was added to XenoRNA and reverse transcribed with SuperPrep (Toyobo Co., Ltd., Osaka, Japan). Real-Time quantitative PCR was performed and the mRNA abundance in each fraction was calculated and normalised by XenoRNA Cq values. The relative RNA abundances were converted to the percent of total detected RNA.

### Animal experiments and cell line xenografts

The animal experiment protocol was approved by the Ethics Committee on Animal Experiments of Nippon Medical School (ethics approval number 26-020, 27-188). It was carried out in accordance with the guidelines for Animal Experiments of Nippon Medical School and the guidelines of The Law and Notification of the Government of Japan^32^, as well as the ARRIVE guidelines. Mice were maintained 12 h light/12 h dark cycle at 20–24 °C with 40–70% humidity. They were allowed to have free access to standard laboratory mouse chow, MF (Oriental Yeast Co., ltd. Tokyo, Japan), and free access to drinking water. They were housed at a maximum number of five. All mice were checked for stress each day. For the xenograft experiments, male BALB/cAJcl-nu/nu were purchased from CLEA Japan, Inc., Tokyo, Japan. Indicated cultured-cells were inoculated in the mammary fat pad of immunodeficient 7 week old male nude mice (body weight 23–26 g) as described^48^. Tumour sizes were measured weekly with a calliper, and tumour volume was determined with the following standard formula: 0.5 × L × W^2^, where L is the longest diameter and W is the shortest diameter. Mice were euthanized by cervical dislocation, then each tumour were removed and weighed, and also collected for further experiments.

### Statistical analyses

All experiments were performed in triplicate unless stated. Data are expressed as the mean ± standard deviation of at least three independent experiments. Statistical analysis of parametric data was performed using the Student’s *t*-test or one-way analysis of variance (ANOVA) with Scheffe’s F test as a post hoc test. Statistical analysis of non-parametric data was performed using the Mann–Whitney *U*-test, or for multiple groups, the Kruskal–Wallis test followed by Steel–Dwass test as a post-hoc test. P-values < 0.05 were considered statistically significant and all p-values are reported with their respective data sets.

## Supplementary Information

Supplementary Information
