# Supplementary material for: MYD88 signals induce tumour-initiating cell generation through the NF-κB-HIF-1α activation cascade
Source: Sci Rep. 2021 Feb 17;11:3991. doi: 10.1038/s41598-021-83603-4 (PMC7890054; doi:10.1038/s41598-021-83603-4)

**SUPPLEMENTARY INFORMATION**

**Title**

MYD88 signals induce tumour-initiating cell generation though the NF-κB-HIF-1α activation cascade

**Authors**

Atsuko Tanimura, Akane Nakazato, Nobuyuki Tanaka

**Supplementary Figures 1-9**


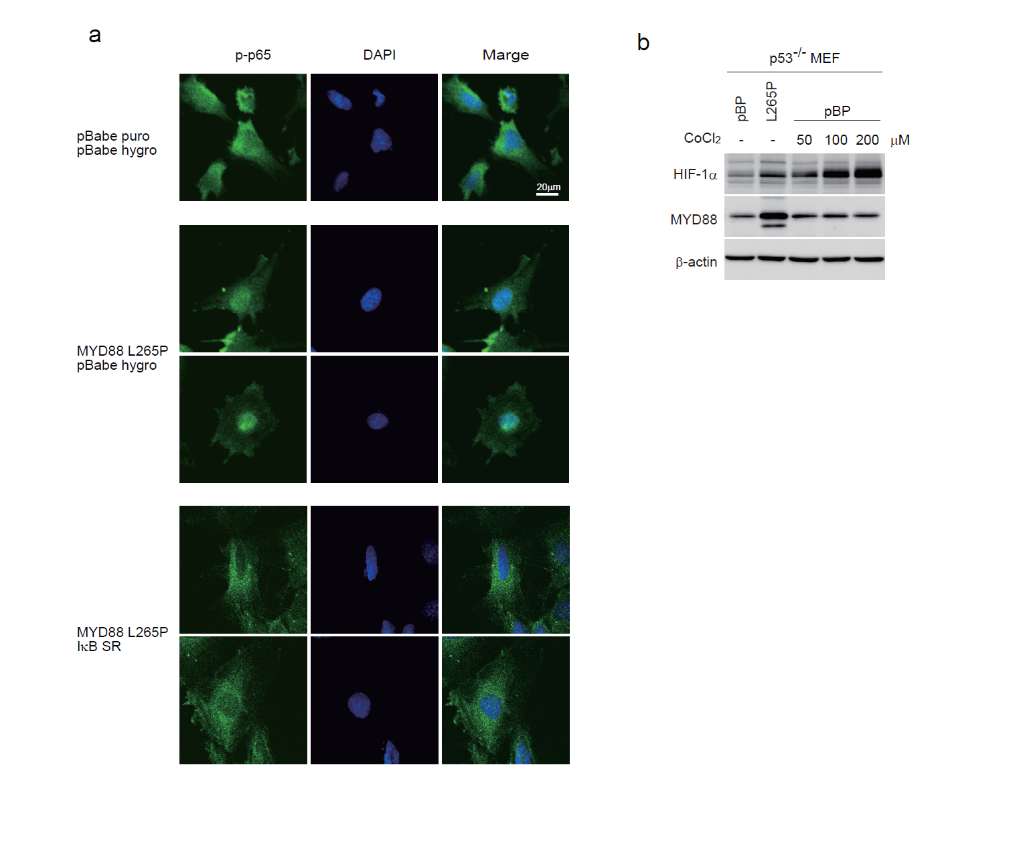


**Figure S1.** MYD88 L265P induces NF-κB p65 phosphorylation and HIF-1α protein accumulation. **a,** Immunofluorescence staining shows phosphorylated NF-κB p65 nuclear localisation in the indicated gene-expressing *p53^−/−^*MEFs. **b,** HIF-1α protein accumulation by expressing MYD88 L265P and cobalt chloride treatment. Total cell lysates were analysed by immunoblotting. Vector introduced *p53^−/−^*MEFs were treated with cobalt chloride for 2 hours.


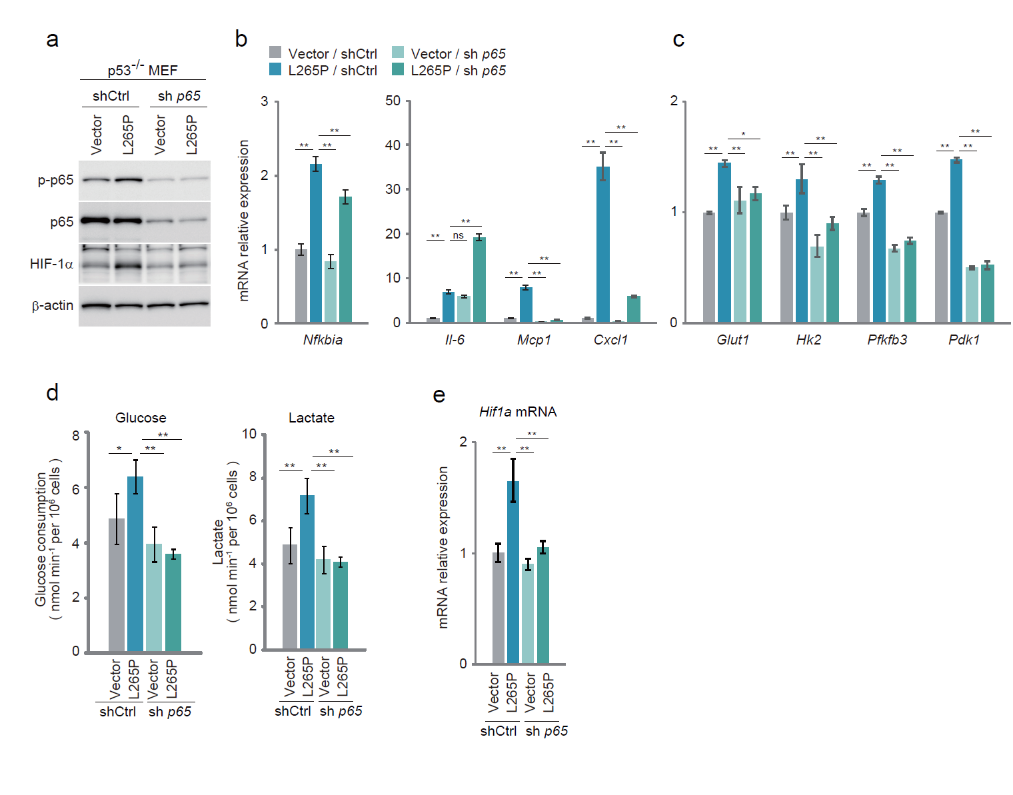


**Figure S2.** Reducing p65 expression by shRNA in *p53^−/−^*MEFs expressing MYD88 L265P. **a,** Total cell lysates were analysed by immunoblotting. **b,** Gene expression of NF-κB targets was measured by qPCR. **c,** Expressions of glucose metabolism-related genes were quantified by qPCR. **d,** Glucose uptake and lactate production were measured (n = 4). The quantified results are presented as the mean ± s.d. using one-way ANOVA followed by Scheffe’s F test. *P < 0.05, **P < 0.01. **e,** Gene expression of *Hif1a* was measured by qPCR. *P < 0.05, **P < 0.01.


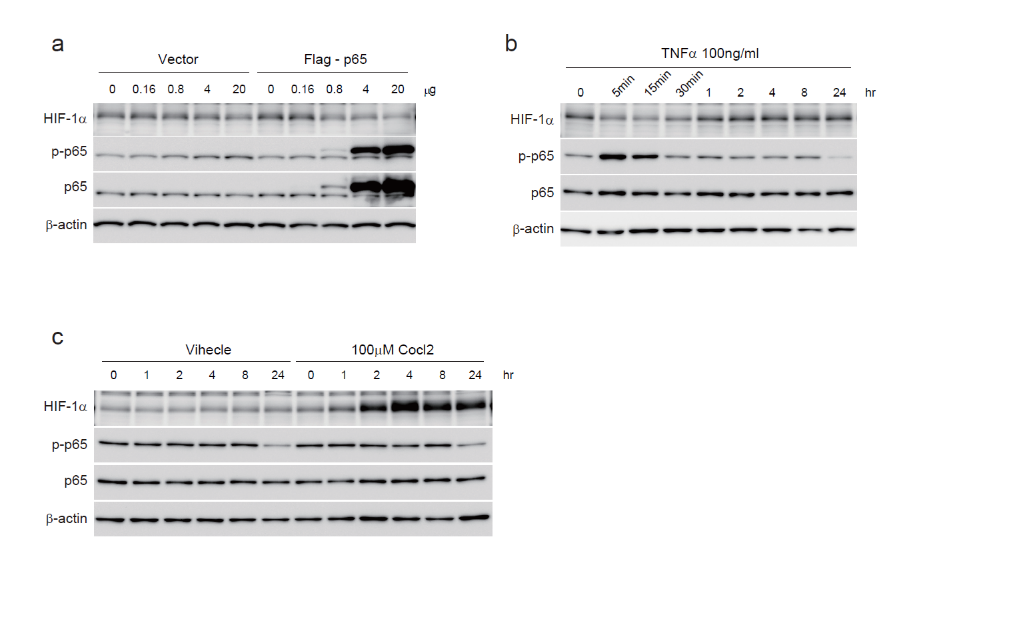


**Figure S3.** NF-κB p65 activation via MYD88 regulates HIF-1α expression in *p53^−/−^*MEFs. Total cell lysate was analysed by immunoblotting. **a,** The indicated amount of vector or p65 construct was transfected into *p53^−/−^*MEFs. **b, c,** *p53^−/−^*MEFs were treated with indicated reagent.


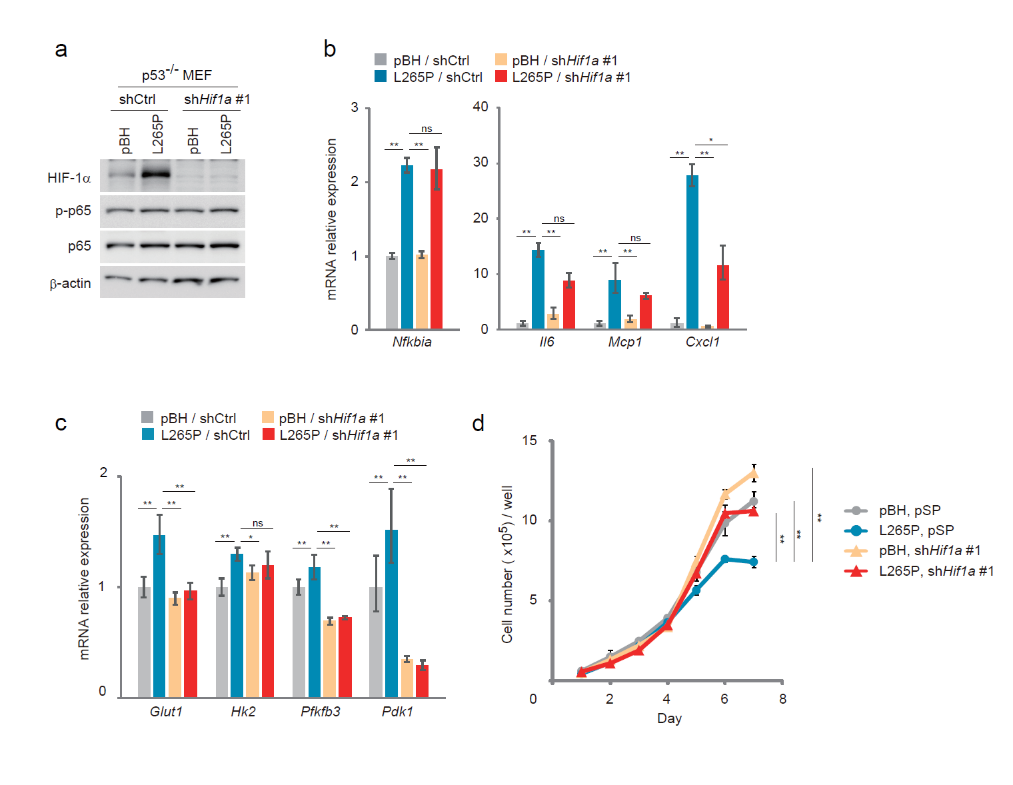
**Figure S4.** Suppression of HIF-1α does not affect NF-κB activation but reduces glucose metabolism in MYD88 L265P-expressing *p53^−/−^*MEFs. **a-d,** Reducing *Hif1a* expression by shRNA (#1) in *p53^−/−^*MEFs expressing MYD88 L265P. **a,** Total cell lysate was analysed by immunoblotting. **b,** Gene expressions of NF-κB targets were measured by qPCR. **c,** Expressions of glucose metabolism-related gene mRNAs were quantified by qPCR. **d,** Cell growth assay was performed using the indicated cells. Cell numbers were counted every day from day 0 to day 7 (n = 3). **b, c,** The Y-axis values are the relative fold change for gene transcripts normalised to β-actin. Data represent the mean ± s.d. (n = 3). *P < 0.05, **P < 0.01.


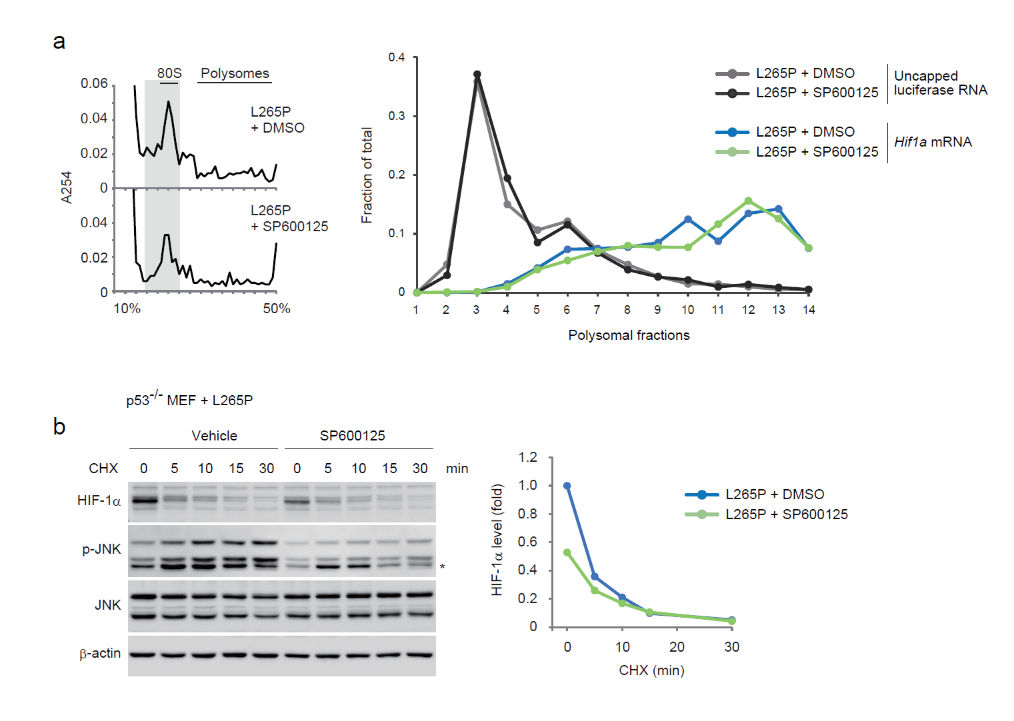
**Figure S5.** The effect of JNK inhibitor on HIF-1α protein translation and stabilisation. **a,** Polysomal fractionation was performed for *p53^−/−^*MEF MYD88 L265P treated with SP600125 to detect *Hif1a* mRNA translation efficiency. Cells were treated 25μM SP600125 or vehicle for 8 hours. The sucrose gradient was 10%–50% and 15 fractions were collected. The OD254 plot for each polysome profiling experiment (left). The relative distribution of Hif1a mRNA associated with each fraction of the gradient was analysed by qPCR (right). **b,** *p53^−/−^*MEFs expressing MYD88 L265P were treated with 25μM SP600125 for 8 hours or vehicle, then treated with 100 μg/ml cycloheximide (CHX) for the indicated times. Total cell lysates were analysed by immunoblotting (left) and the quantification of HIF-1α signals is shown (right). The asterisk indicates a nonspecific band, since it is lower than endogenous JNK band.


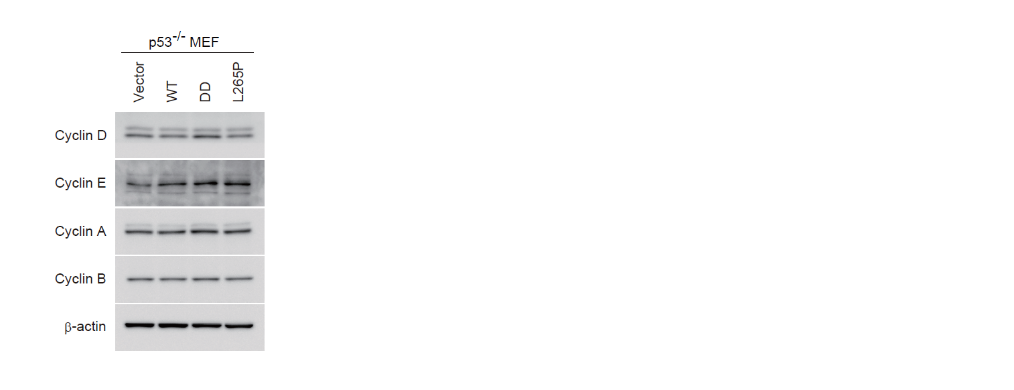


**Figure S6.** MYD88 L265P-expressing *p53^−/−^*MEFs form tumours via the NF-κB-HIF-1α axis. Protein expression of cell cycle regulated factors in cultured MYD88 L265P-expressing *p53^−/−^*MEFs was analysed by immunoblotting.


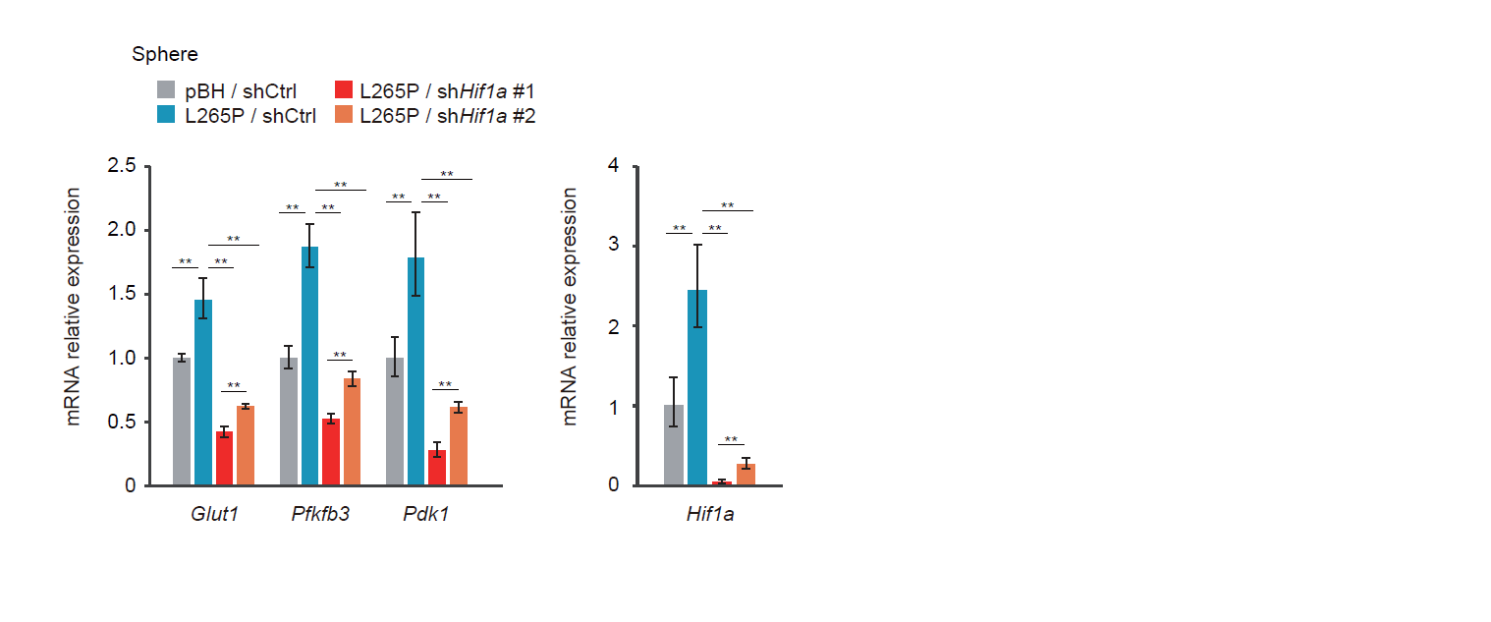
**Figure S7.** The efficiency of sh*Hif1a* #1 and #2 in spheres of *p53*^−/−^ MEFs expressing MYD88 L265P. Expressions of glucose metabolism-related genes *Glut1*, *Pfkfb3*, *Pdk1*, and *Hif1a* were quantified by qPCR in sphere cells from MYD88 L265P-expressing *p53^−/−^* MEFs expressing sh*Hif1a* #1, #2, or sh control (shCtrl). The y-axis values represent the relative fold change of gene transcripts normalized to β-actin. Data represent the mean ± s.d. (n = 3). *P < 0.05, **P < 0.01.


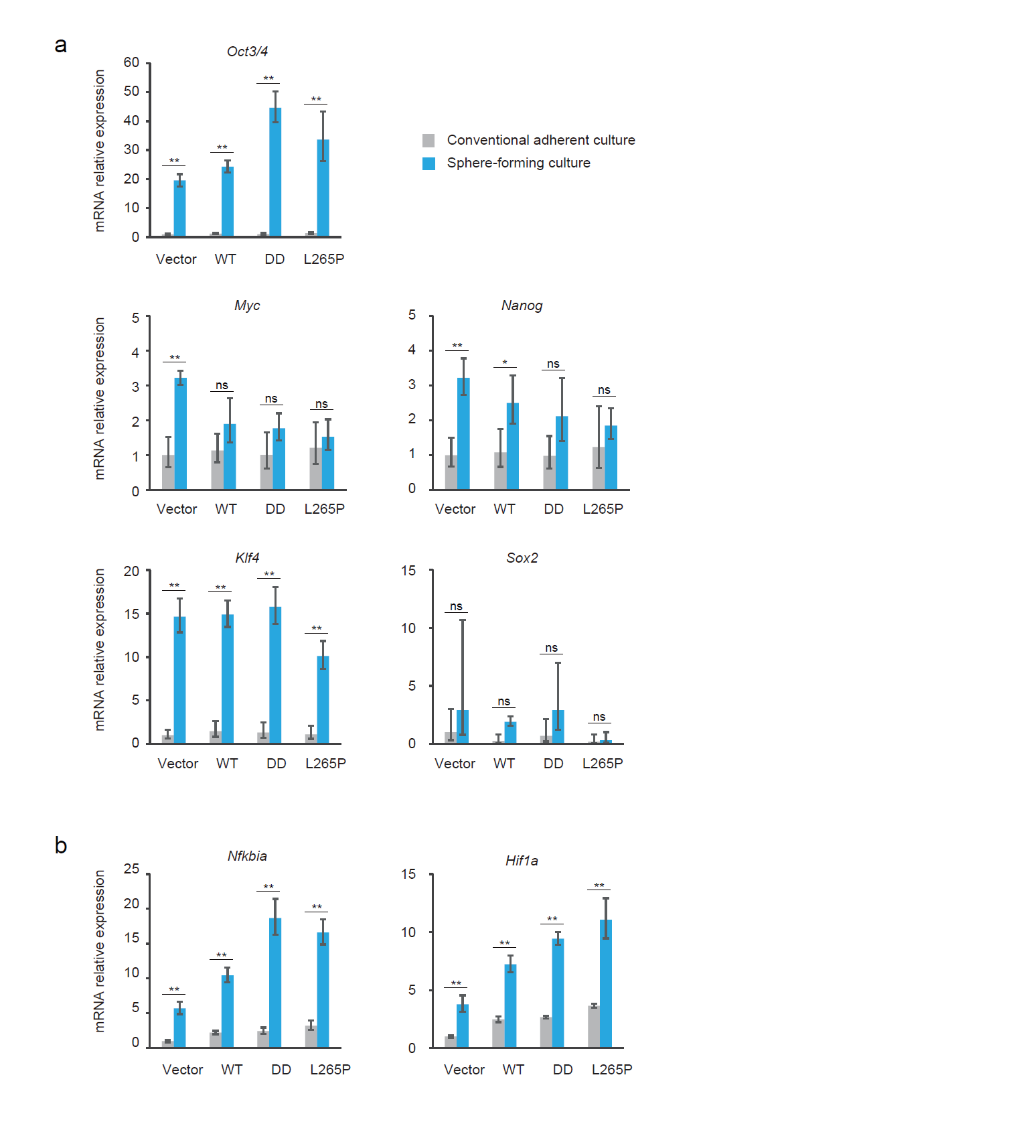


**Figure S8.** Comparison of mRNA expression in spheres and in adherent cells from MYD88 L265P-expressing *p53^−/−^*MEFs. **a, b,** Expressions of the indicated gene mRNAs were quantified by qPCR. The Y-axis values are the relative fold change for gene transcripts normalised to β-actin. Data represent the mean ± s.d. (n = 3) using one-way ANOVA followed by Scheffe’s F test. *P < 0.05, **P < 0.01.

**Figure S9.** The images of the original blots for each figure are shown below.


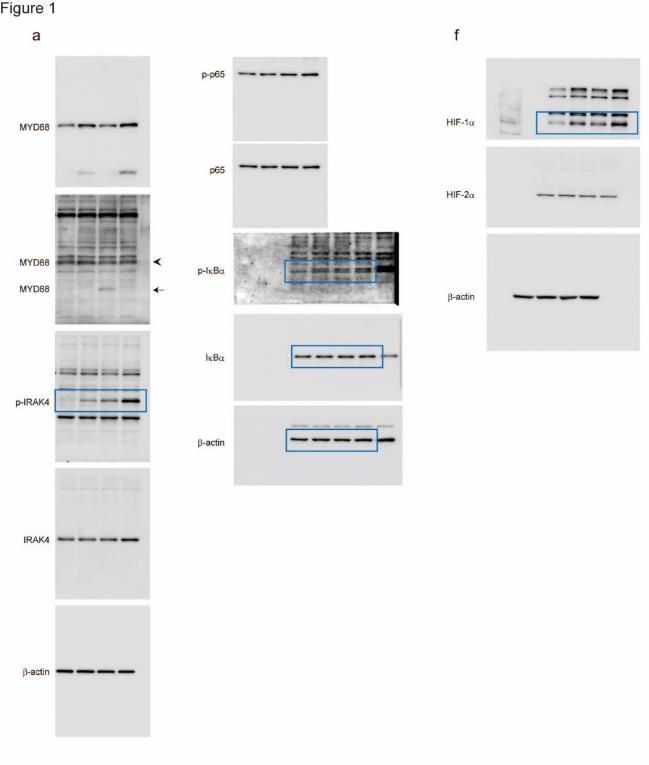


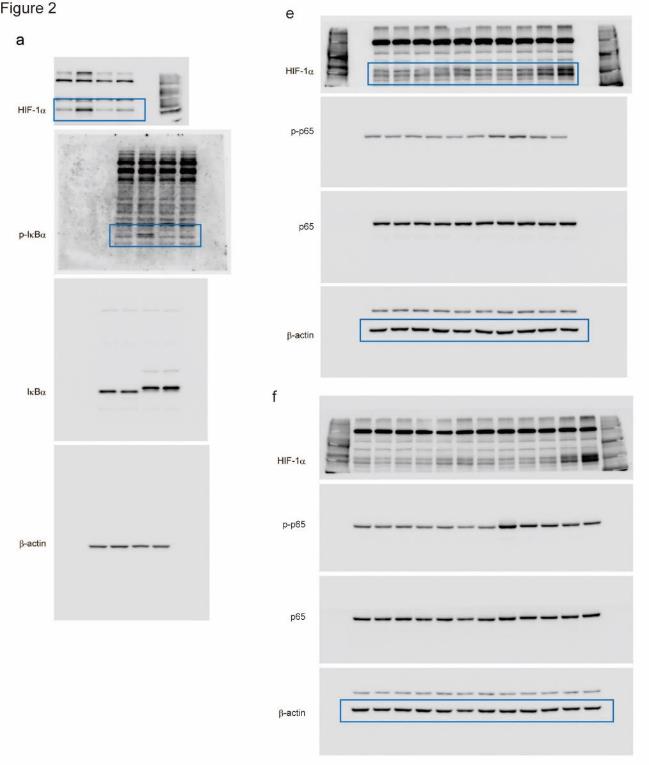


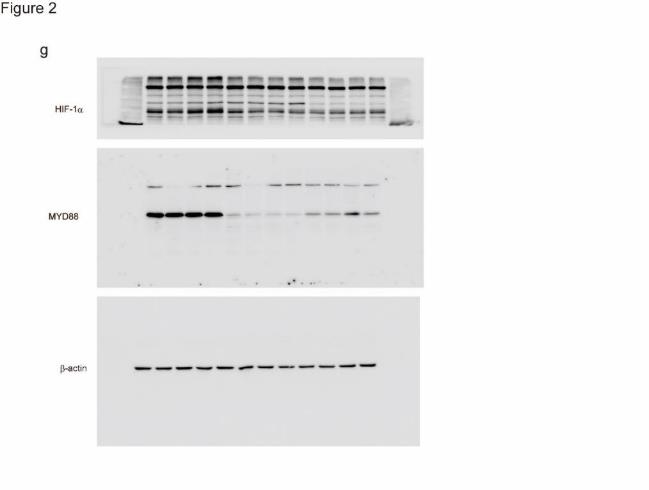


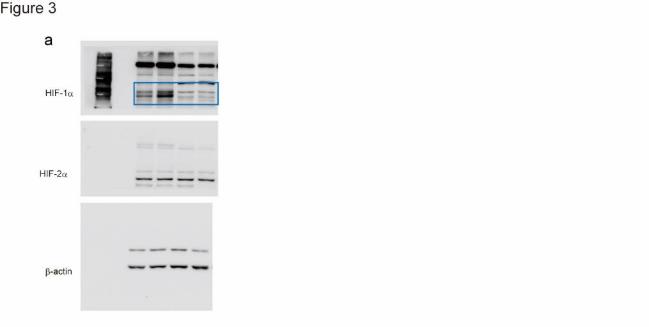


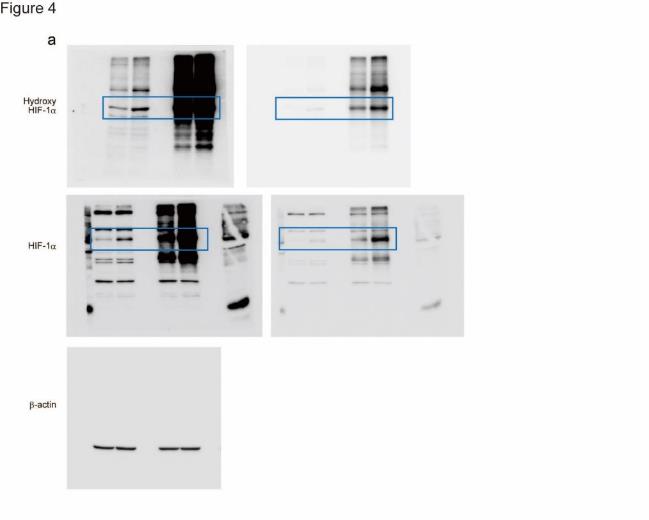


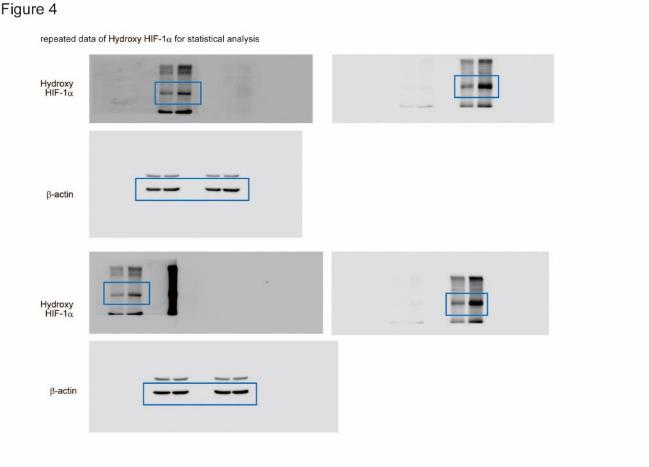


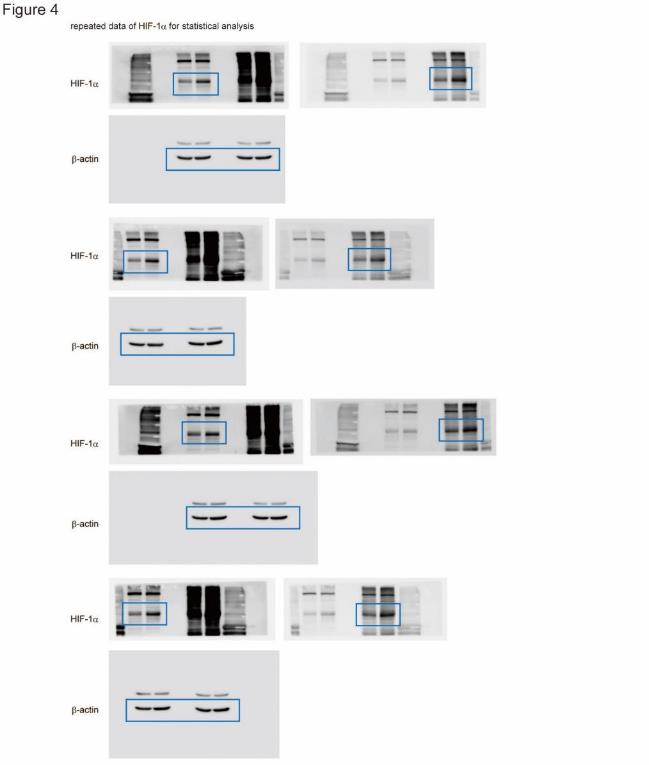


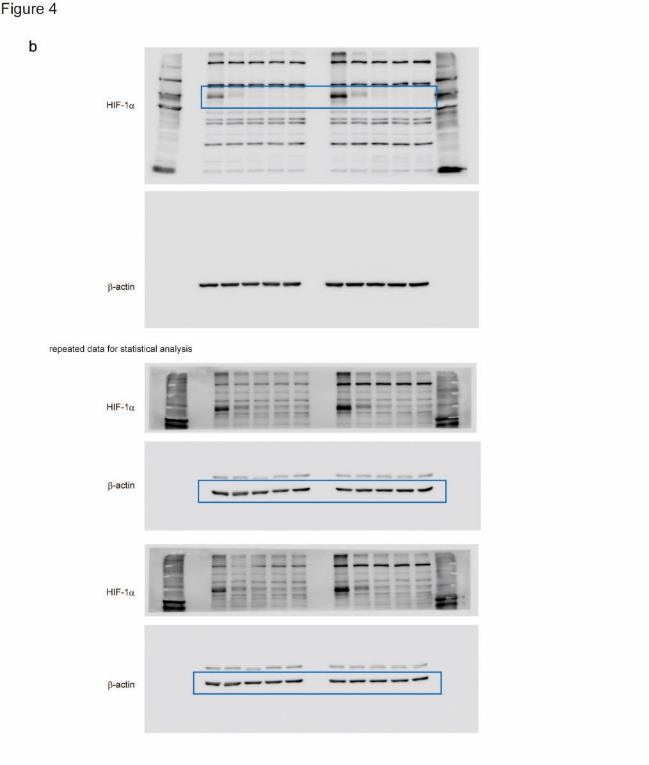


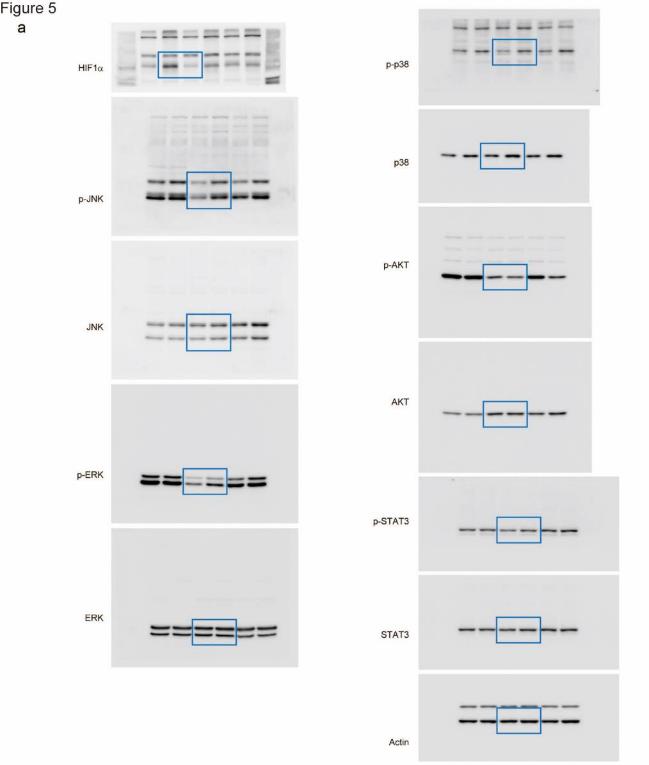


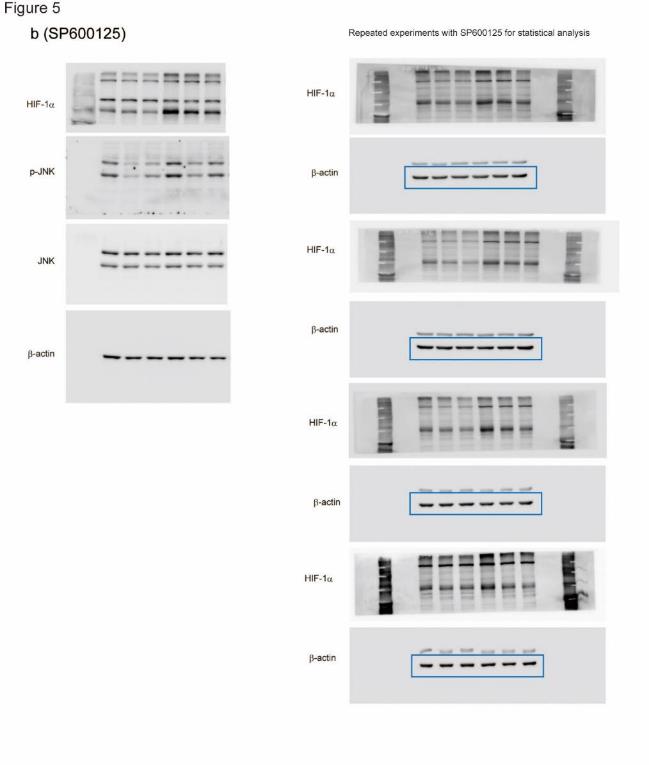


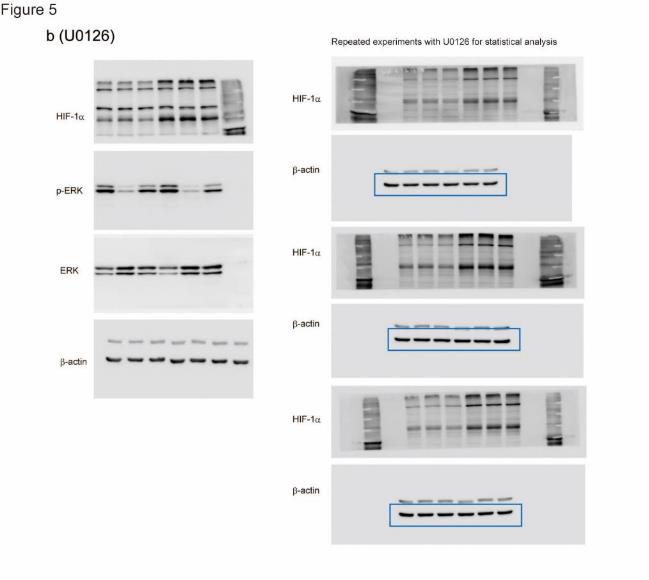


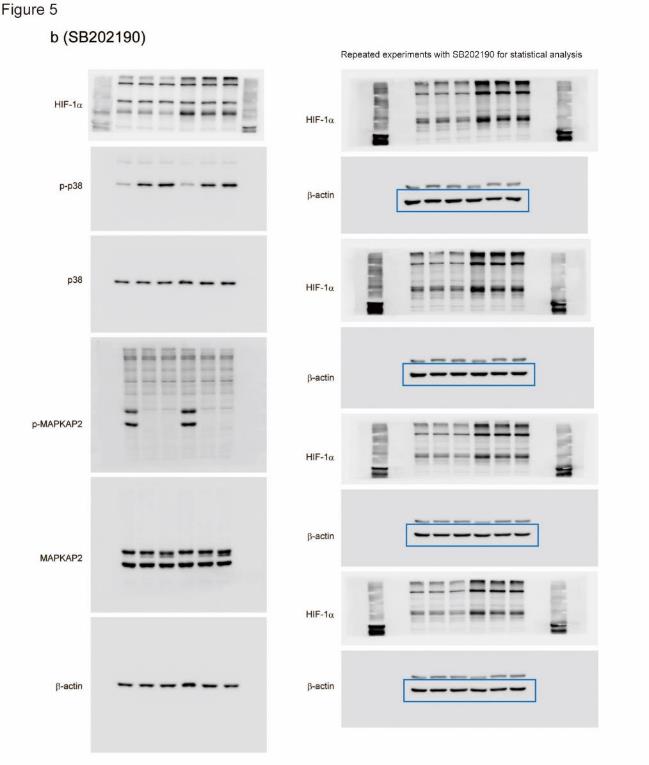


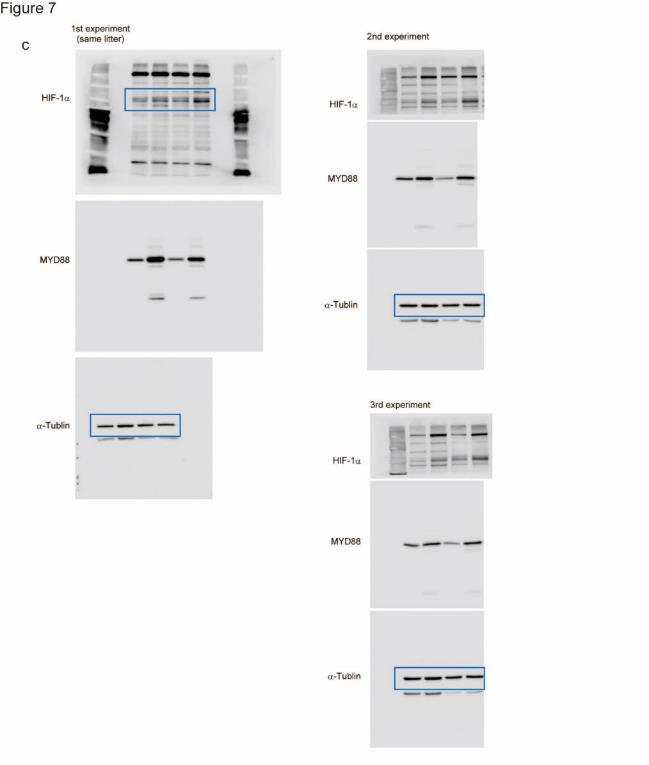


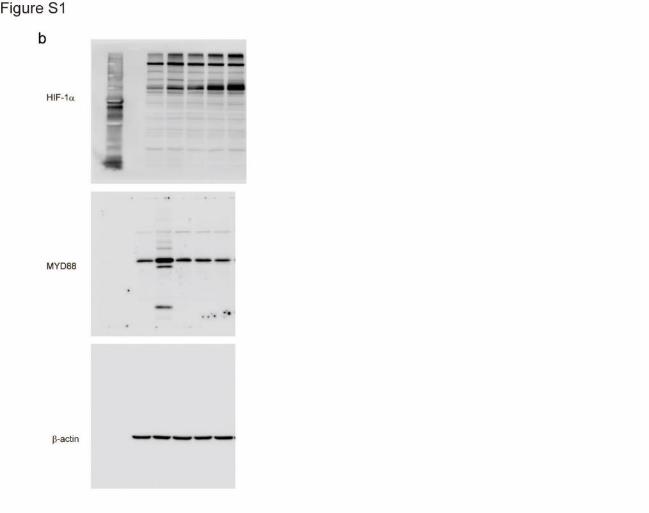


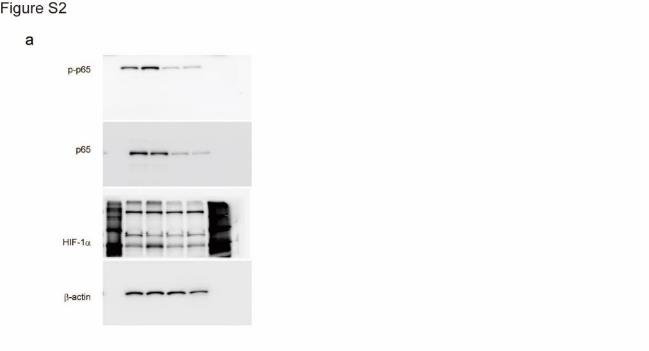


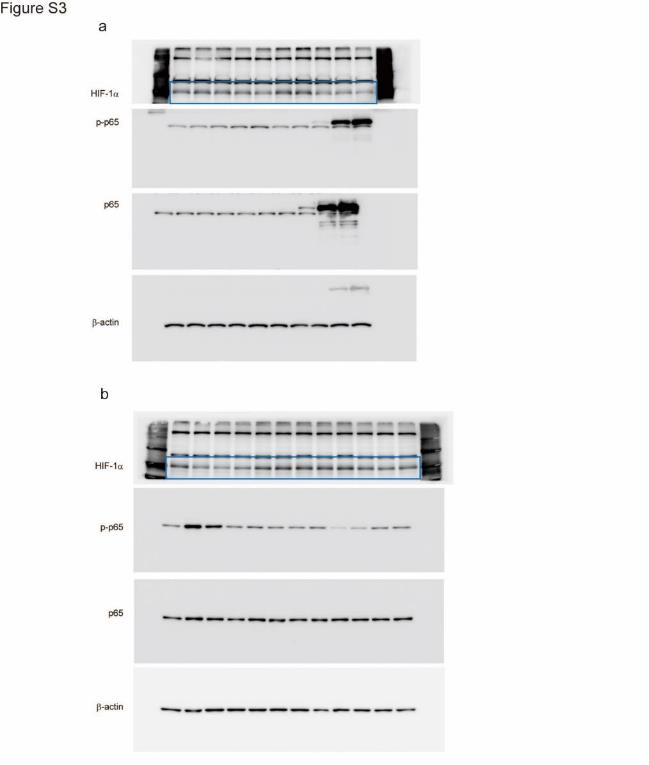


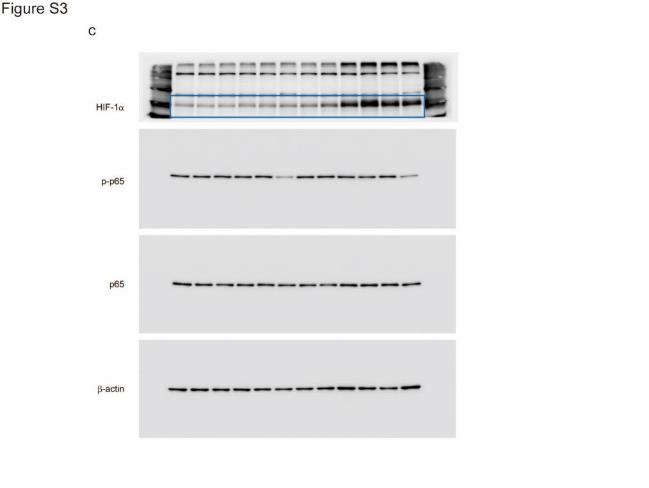


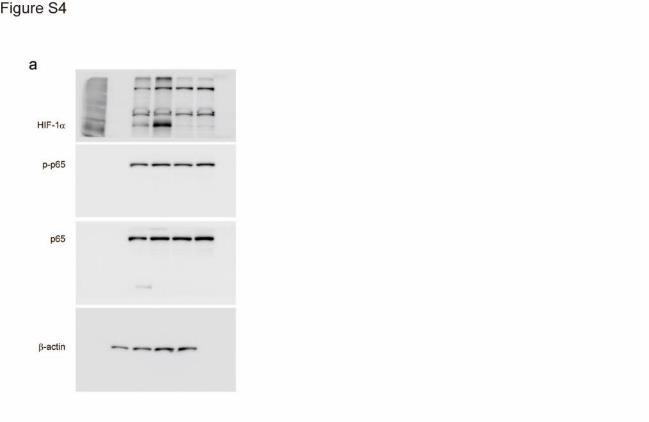


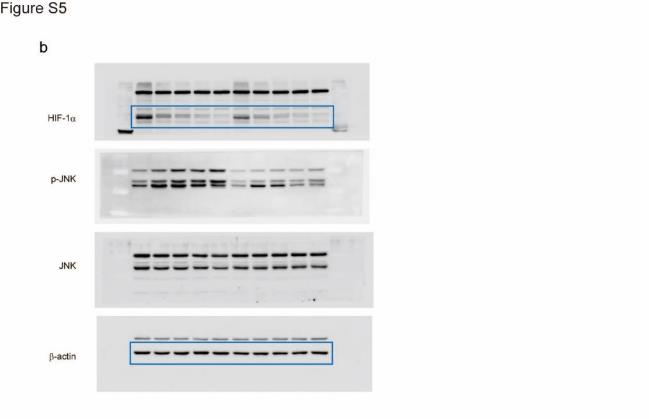


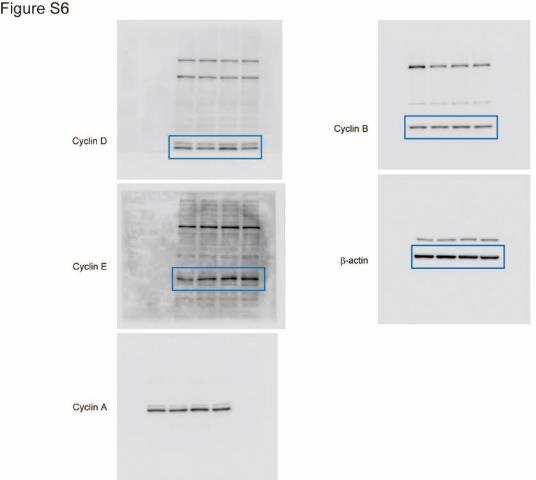

Supplement: Supplementary file 1 — Supplementary Information [file 41598_2021_83603_MOESM1_ESM.docx]
